# 10,000 years of centennially-resolved climate and sea-level change archived in Svalbard beach-ridge system

**DOI:** 10.1038/s41598-025-33652-w

**Published:** 2026-01-03

**Authors:** Mateusz C. Strzelecki, Sebastian Lindhorst, Christopher J. Hein, Willem G. M. van der Bilt, Katherine E. Kivimaki, Jan Kavan

**Affiliations:** 1 Alfred Jahn Cold Regions Research Centre, Institute of Geography and Regional Development, University of Wrocław, pl. Uniwersytecki 1, 50-137 Wroclaw, Poland; 2 Institute for Geology, Center for Earth System Research and Sustainability, University of Hamburg, Bundesstr. 55, 20146 Hamburg, Germany; 3 Virginia Institute of Marine Science, William & Mary, Gloucester Point, VA 23062 USA; 4 Department of Earth Science and Bjerknes Centre for Climate Research, University of Bergen, Bergen, Norway; 5 Centre for Polar Ecology, Faculty of Science, University of South Bohemia, České Budějovice, Czechia

**Keywords:** Uplifted beach-ridges, Relative sea-level, Arctic coastal evolution, Arctic paleoclimate, Ground-penetrating radar, Geomorphology, Cryospheric science, Palaeoclimate

## Abstract

Over the last 10,000 years, no fewer than 178 beach-ridges were formed and preserved at Bjonasletta in central Spitsbergen, recording shifts in relative sea-level and fluctuations in glacier- and sea ice extent. Shell-based dating of beach-ridge crests combined with a centimetre-scale photogrammetry-based digital elevation model reveal that the formation of this beach sequence progressed through three major phases of relative sea-level fall. In line with other records from the central part of the archipelago, we observe a rapid (0.9 m per century) fall in sea-level during the Early Holocene, followed by a gradual deceleration (0.45 m per century) during the Middle Holocene, until near-modern levels were reached around 2.2 ka. Ground-penetrating radar surveying of the internal sedimentary architecture of the beach-ridges suggests that plain progradation and ridge formation were dominated by continuous swash accretion under fair-weather conditions. We argue that climate changes are recorded by the geometry and formation rate of individual ridges. Correlations of the beach-ridge record with independent climate proxy data suggest that warmer conditions at 10–6 ka were favourable for supplying the beach-ridge plain with sediments from nearby slopes and reworked glacial landforms, allowing ridges to form at a relatively rapid rate of ~ 45 yrs per ridge. Climate cooling, particularly after 4 ka during the Neoglacial period, allowed for the expansion of sea-ice coverage in the fjords and the advance of marine-terminating glaciers, which reduced fetch in the fjord and limited the transfer of wave energy onto the Bjonasletta beach. This led to a reduction in sediment supply to the beach-ridge plain and an attendant slowing of progradation to ~ 100 yrs per ridge. The Neoglacial was further characterized by the development of coastal permafrost across the beach-ridge plain, which crept down from extensive talus slopes and dissected ridges through frost fissure and ice-wedge polygons. Our findings suggest that the morphology and growth patterns of beach-ridge plains, common throughout global high latitudes, can archive signals not only of sea-ice and sea-level fluctuations, but also of glacial advance and retreat, opening a new avenue for paleo-environmental reconstructions in the planet’s most sensitive and rapidly changing regions.

**Supplementary Information:**

The online version contains supplementary material available at 10.1038/s41598-025-33652-w.

## Introduction

Beach-ridge systems are well-established geological archives of long-term Holocene climate change, sea-level fluctuations, and events^1–4^. Flights of well-preserved raised beaches and beach-ridge sequences are emblematic of Arctic coastlines, where sea-level has dropped since deglaciation, and have been used as proxies for various climatic parameters such as storminess, wave climate, sediment supply, and the pace of glacio-isostatic adjustment^5–17^.

In recent decades, high-latitude beach-ridge records were also used for the reconstruction of sea-ice variability, for example, in northern Greenland^14^ and the central part of the Canadian Arctic Archipelago^13^. In Svalbard, Long et al.^18^ demonstrated that selection of storm-deposited, articulated specimens of juvenile molluscs from the beach crests (highest point of a beach where wave action deposits material) can avoid the problem of dating reworked shells, improving chronological controls on beach deposition and relative sea-level change. Lindhorst & Schutter^19^, using ground-penetrating radar (GPR) on beaches in Antarctica, showed that the internal sedimentary architecture of polar beach-ridges depends on the local wave climate and that long-abandoned ridges at higher elevations can be subject to reactivation. In addition, Nielsen et al.^16^, working along the western and southern coast of Greenland, demonstrated that GPR surveying of beach-ridges can yield precise sea-level index points that are consistent with respect to the influence of vertical land movement.

Svalbard is a critically important region for research into high-latitude Holocene climate and landscape change, owing to its unique geography that bridges Arctic and Atlantic influences, and its rich geological records that record high-amplitude climate changes such as meltwater floods like the 8.2 ka event^20^, the onset of Neoglaciation during the Late Holocene^21^, as well as the Little Ice Age^22^. The wealth of uplifted beaches in this region are established sources of information on past sea -level and the related spatio-temporal pattern of ice sheet and glacier decay or advance^8,10,18,23–28^. Reliable sea-level curves of Svalbard are based on (a combination of) mapping beach surface morphology, identification of geochemically distinct pumice, and radiocarbon dating of driftwood and whalebones^27,29–35^. Yet few researchers harness the potential of these records to capture changes in climate, coastal processes, or coastal landscapes preserved within their internal architecture or surficial morphology. Additionally, chronological control is often insufficient to constrain and contextualize Arctic coastal changes on human-relevant (centennial or shorter) timescales. It should also be emphasized that one of the long-standing challenges in establishing an accurate sea-level datum in the Arctic has been the lack of precision in elevation measurements. This paucity of research has precluded the analysis of fine-scale (cm-scale) topographic changes in beach-ridge morphologies, as well as the determination of the precise elevation of samples used to establish geochronologies. The integration of Unmanned Aerial Vehicle (UAV) technology into Digital Elevation Model (DEM) construction has the potential to address this gap.

Our study site the Bjonasletta beach-ridge system is strategically located in central Spitsbergen, major island of Svalbard, at the approximate hinge point between deglacial land emergence and submergence^36^. We employ high-resolution UAV imagery and combine these data with GPR surveys, morphometric analyses of centimetre-scale beach-plain topography, and centennial-scale radiocarbon dating of well-preserved juvenile mollusc shells. Our study assesses the sensitivity of the beach-ridge plain to relative sea-level change, and reveals the potential of these archives to record shifts in climatic conditions that triggered glacier and permafrost evolution, sea-ice extent, as well as sediment supply.

## Setting

**Fig. 1:**
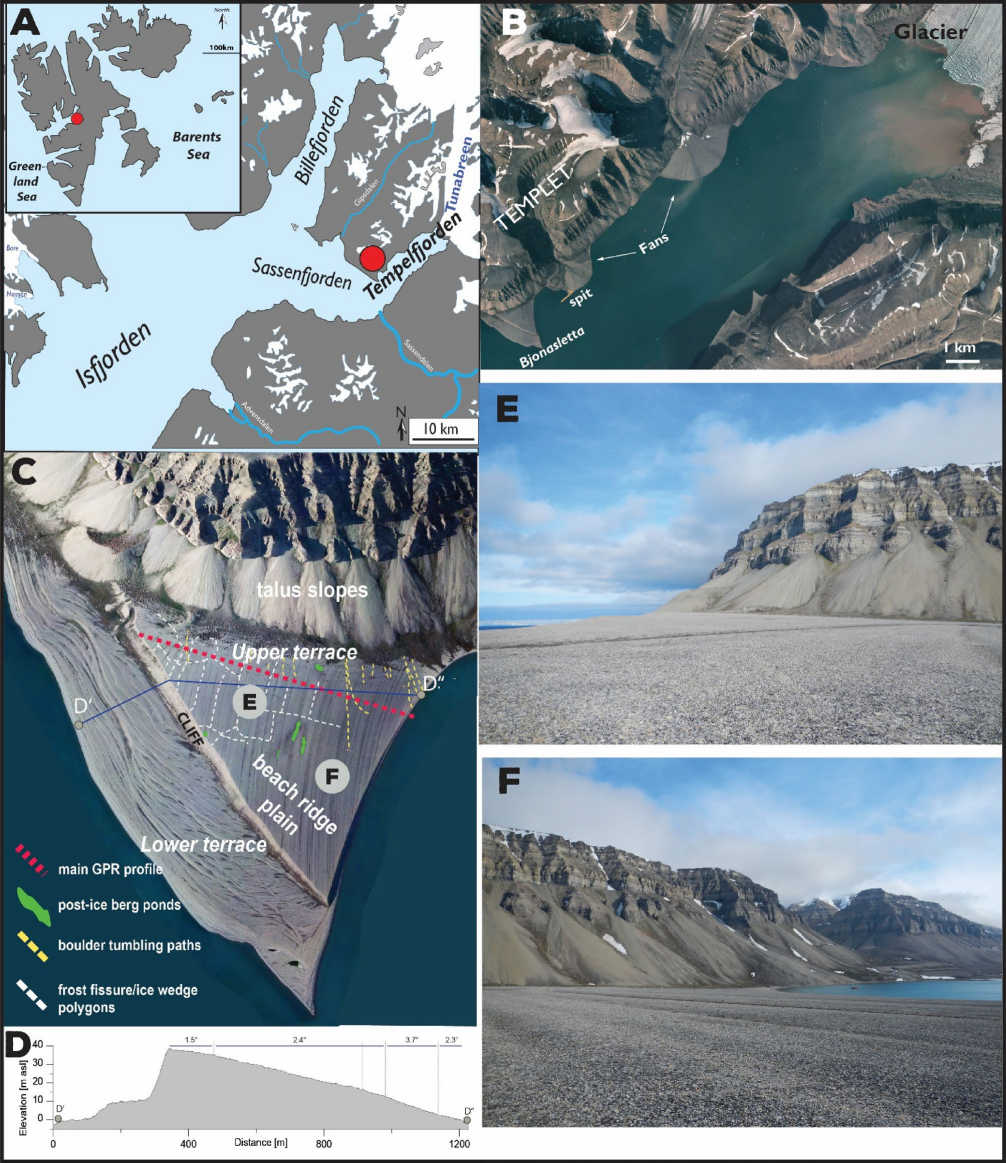
(**A**) Location of our study area in central Spitsbergen, the main island of the Svalbard Archipelago. Bjonasletta is situated at the junction of Sassenfjorden and Tempelfjorden in the inner part of Isfjorden; (**B**) Major elements of the coastal landscape in Tempelfjorden: Bjonasletta beach-ridge plain, alluvial fan deltas, and the marine-terminating glacier Tunabreen.© Norwegian Polar Institute. The aerial photograph is used with courtesy of the Norwegian Polar Institute. From https://toposvalbard.npolar.no. (**C**) The Bjonsletta beach system. Note the morphological difference between the upper and the lower terraces divided by a ~ 11–13-m high cliff. On the surface of the upper terrace, a network of frost fissures has developed (white dashed lines); green polygons indicate terrain depressions in the surface of the beach-ridge plain associated with iceberg deposition. Grey circles with letters indicate the locations of the photos (**E**) and (**F**). The aerial photograph in the background is used with courtesy of the Norwegian Polar Institute. (**D**) Topographical profile along line D’-D” crossing the lower and upper beach terraces. (**E**) wide beach-ridges formed during the Early Holocene; (**F**) narrower beach-ridges developed since the Late Holocene. The figure was created in Adobe Illustrator 2025 https://www.adobe.com).

The beach-ridge plain of Bjonasletta is located at the confluence of the Sassen- and Tempelfjorden, the north-eastern branches of Isfjorden, the largest fjord system of Spitsbergen (Fig. 1A). The beach-ridge plain has a triangular shape with the longest side facing southwest and edge lengths of approximately 1.9 × 1.5 × 1.3 km. The plain is northward-attached to the mountain complex of Templet with a textbook example of a cold-region talus slope system^37^. Two large active fan deltas are located northeast of the Bjonasletta beach-ridge plain; at distances of one and five kilometers, respectively (Fig. 1B). The age of these fan deltas is unknown. However, the one more proximal to the beach plain intersects marine terraces at elevations similar to the highest point of the Bjonasletta beach-ridge plain. A marine terminating glacier, the Tunabreen, is located at the easternmost termination of Tempelfjorden. Reconstruction of the decay of the Svalbard-Barents Sea Ice Sheet suggests that Tempelfjorden maintained this marine-terminating glacier system throughout the entire Holocene^38^. During the Early Holocene (before ~ 11.2 ka BP), ice-stream retreat left a recessional moraine at the confluence of the fjords, which formed the structural anchor of the Bjonasletta beach-ridge plain^38^.

This pattern agrees with the results of numerical modelling of the wave climate west of Svalbard, which shows that swells approaches the island predominantly from the southwest while wind-waves come from northwesterly to southwesterly directions and from the northeast^40^. The comparison of different reanalysis data sets covering the period 1981 to 2010 also underlines a distinct seasonality in Svalbard storm climate, with stronger storms being more frequent in winter, and usually forming in the Barents Sea^41^ (Fig. 1). Most recent observations indicate a substantial decline in wave attenuation rates in the vicinity of Svalbard, transitioning to a state of reduced ice coverage. This decline enhances the propagation of wave energy and diminishes the ice’s intrinsic dampening effect on waves^42^. Specifically, the lack of sea ice over the last two decades has been demonstrated to lead to a substantial increase in wave energy reaching the coastal zone. Herman et al.^43^ quantified this change, reporting an increase of up to 100% in wave energy between February and April, coupled with a critical shift in the seasonal maximum of this energy from late autumn (November–December) to the deeper winter months (December–March). Overall, these changes signify a heightened exposure of the coasts to wave-induced erosion and sediment transport^43,44^. The first multi-year, continuous wind wave and water level dataset collected in the Hornsund fjord, southern Svalbard (with much bigger exposure to oceanic swell and storms developing in Greenland Sea than our study site), between 2013 and 2021, reported a distinct spatial gradient in mean significant wave height within the fjord system with wave heights ranging between 1.2 m and 1.3 m in the mouth, 0.5 m and 0.9 m in the central part and less than 0.4 m in the inner fjord^45^. While the mean waves are relatively small, their study and other reports^46^ highlighted that the 99th percentile (the highest 1% of waves) for the open ocean west of Svalbard can reach 5 to 6 m, illustrating the large difference between the open sea and the protected fjord environment.

Marine sediment core and seismic data from the adjacent Isfjorden suggest the area became ice-free from 14 ka BP onwards, during a stepwise deglaciation^47^. Between around 11.7 and 8 ka BP, during the early part of the current Holocene interglacial, conditions were as warm as, or even warmer than, present, as a combination Atlantic water flow and high summer insolation led to warmer climatic conditions^48,49^. Following this optimum, Svalbard climate experienced a series of cooling steps towards the onset of the Neoglacial period around 4 ka BP^50^. Climatic deterioration was accompanied by an increase in ice rafting and glacial sediment input as glaciers advanced into the fjord^49^.

The Bjonasletta beach system comprises two morphological units (Fig. 1C): First, an upper beach-ridge plain (this study), which slopes by up to 3.7° towards the east-northeast (the interior of Tempelfjorden), from 45 to 1 m above modern mean sea- level (asl), and comprises 178 pairs of near-parallel ridges and swales. Secondly, a lower beach system composed of several beach terraces below 12 m asl, which border the beach-ridge plain along its southwest and southerly margins, i.e., facing the open Isfjorden. These terraces have a complex morphology, comprising multiple truncated and intersected spits and berms that accumulated alongshore from north to south, as well as sea-ice push and melt-out features. The upper beach-ridge plain and lower beach terraces are separated by a cliff up to 13 m in height, which truncates the beach-ridge plain oblique to the dominant ridge dip direction and, as a result, lowers in elevation towards the southeast (cliff height at the southern end is 11 m asl).

## Results and discussion

### Surface morphology

The upper beach-ridge plain of Bjonasletta dips towards the east, i.e. the inner part of Tempelfjorden, and is characterized by a low ridge-and-swale morphology (Fig. 1). Individual ridges have amplitudes of up to 0.5 m above adjacent swales; they are slightly curved, and strike NNE-SSW in the oldest (upper) part of the sequence and NNW-SSE in the younger (lower) part.

Surface sediment is composed of angular carbonate clasts ranging in size from coarse granules to cobbles, likely derived from erosion of the adjacent cliffs of Templet (Carboniferous limestones with dolomites and marls, cherts, and shales). The western part of the beach-ridge plain exhibits a pattern of surface fissures, which resemble thermal contraction crack polygons, typical for periglacial environments, and found on the surface of many Svalbard sand and gravel raised beaches. These are likely intermediate fissure polygons^51^ and are clearly visible in the field as shallow (0.1–0.2 m deep) depressions cross-cutting the ridges and swales, as well as on the aerial images, where they are traceable over several hundreds of meters (longest measured crack was 360 m, transecting beach-ridges between 30 and 14 m asl) (Fig. 1C). Conditions favorable to the development of fissure polygons occurred in the Late Holocene, after 4 ka, with the arrival of a cooler and drier climate^52^. This coincides with the onset of permafrost development on uplifted terraces in Adventdalen (western Isfjorden) around the same time^53^. Another characteristic surface feature of the plain are sets of impact scars associated with the tumbling of boulders likely originating from rockfalls from Templet. Scar paths are up to ~ 400 m long (Fig. 1C). Finally, several dry depressions and small ponds (Fig. 1C) are found in the central and southern parts of the foreland, which we tentatively attribute to the stranding and subsequent melting of icebergs^54^.

### Sedimentary architecture and genesis of beach-ridge plain

**Fig. 2:**
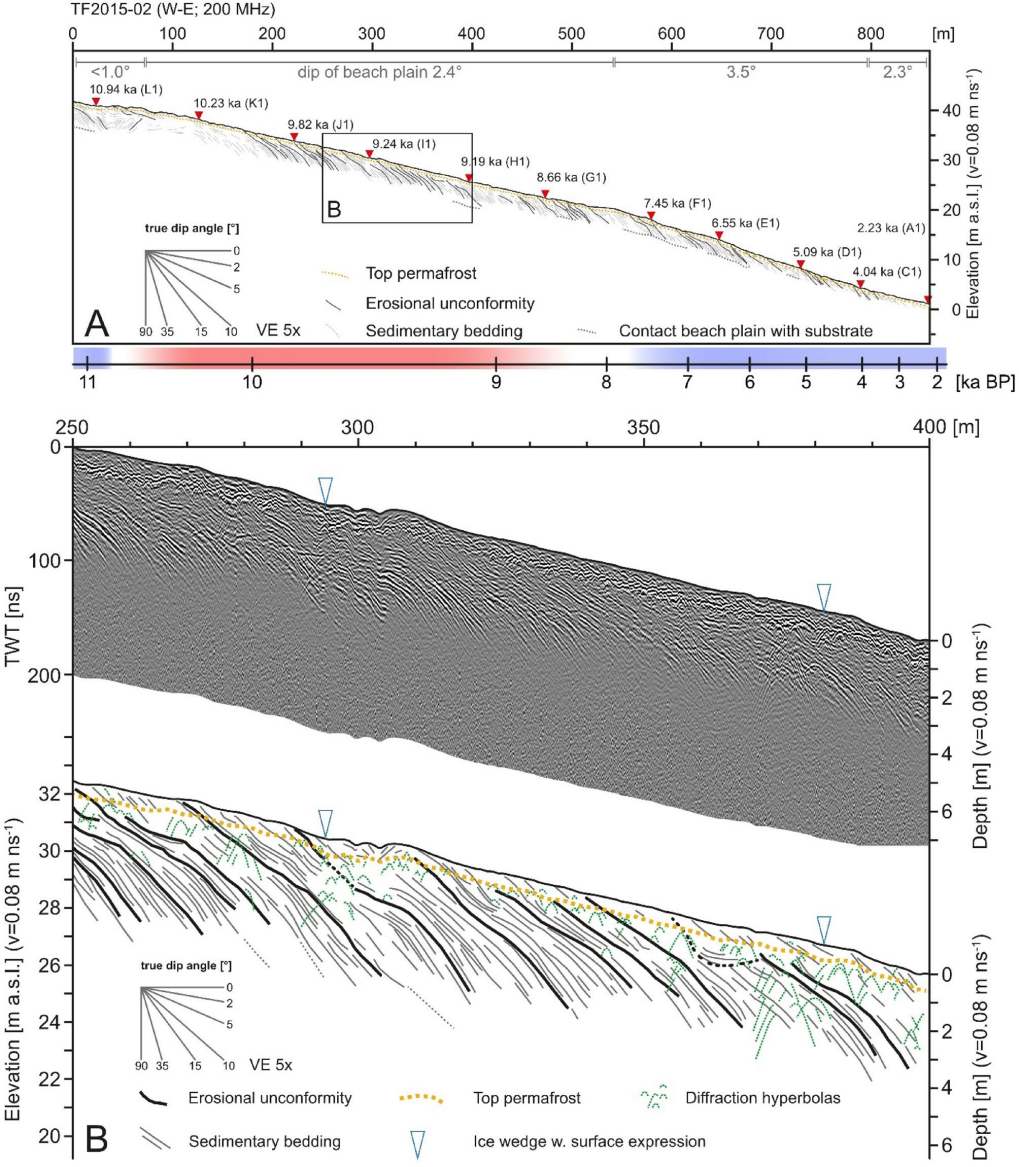
(**A)** Interpretation of sedimentary geometries along GPR line TF2015-02. See Fig. 1 for location of line and Supplementary Material 1 for GPR data of complete line; ages are radiocarbon ages (see Table 1); yellow and blue bars indicate warm and cold climate episodes, respectively (compare Fig. 6); (**B)** Detail of GPR line TF2015-02 from 250 to 400 m with interpretation (see A for location).

Nineteen GPR lines with a total length of 9 km (see Methods and Materials section for survey details), allow for mapping of the internal geometries of the upper beach-ridge plain. The 860 m long, WSW-ENE striking line TF2015-02 cross-cuts the sequence of beach-ridges in a proximal position, near the contact of the beach plain with the lower slope of Templet (Figs. 1 and 2, Suppl. 1). Ground penetration decreases from 3 to 5 m in the west of the line to about 1 m near the eastern termination of the line. Reflection amplitudes are high in the uppermost meter of the succession and between ~ 2–4 m below the surface, whereas reflections in between these two intervals are weaker and appear less continuous. Diffraction hyperbolas, likely caused by cobbles and boulders in the subsurface, are common throughout the dataset but seem to cluster between 0.5 and 1 m below the surface (Fig. 2B). Vertically stacked diffraction hyperbolas mark the downward continuation of the permafrost-related polygonal surface cracks that intersect the plain.

Beach-plain stratigraphy is dominated by eastward-dipping beds of tabular to slightly sinusoidal shape. Sedimentary beds dip at up to 10° down to depths of 1.5–2.0 m below the surface, less steep (~ 5°) between ~ 2 and 3 m, and up to 15° at greater depths. Numerous individual beds can be traced laterally for several tens of meters along the line and from the surface to depths exceeding 4 m. The contact between beach-plain sediments and the underlying substratum is very weak and discontinuous reflection in the GPR data (Fig. 2A; Supplementary Material 1). From the data available, we find that Bjonasletta beach sediments are > 5 m thick, exceeding other high-latitude gravel beaches, which are often < 2 m thick^19,55^. Erosional unconformities intersect the sediments of the prograding beach plain (Fig. 2). In most cases, these are not imaged as a distinct reflection in the GPR and are only detectable by reflection truncations. The dip of erosional unconformities is similar to that of the beach strata; their shape, however, is less curved.

The predominance of seaward-dipping strata, as well as the gentle ridge and swale surface morphology, resemble the architecture of gravel beach-ridge plains described from sheltered coastal segments in Antarctic^19^. The architecture of those Antarctic ridges, however, comprises storm-built beach-ridges which unconformably overlay a fair weather-prograding beach plain. Such a distinct partitioning of process seems to be missing at Bjonasletta. Here, ridge-internal strata are traceable through the beach plain and there is no unconformity between ridges and the prograding beach. This architectural style indicates that individual ridges were formed synchronous to beach-plain progradation and do not represent architectural elements formed under higher-energy waves^19^. A storm-dominated genesis of the Bjonasletta ridges therefore seems unlikely.

### Geochronology and sea-level record

Beach-ridges within the upper Bjonasletta plain range in age from 10.8 to 2.2 ka BP (see Table 1 for details on beach-ridge chronology). The crest of the active berm at the modern beach is at 1.5 m above present mean tide level, which was calculated using the chart datum established for nearby Longyearbyen port (available at: https://www.kartverket.no/en/at-sea/se-havniva/result?id=966478location=Longyearbyen. Our subsurface data demonstrates that these ridges are mild swash-built, and not storm ridges. Given this, we interpret each ridge crest as having formed at 1.5 m Mean Sea Level (“indicative meaning”), providing sea-level index points.

**Fig. 3:**
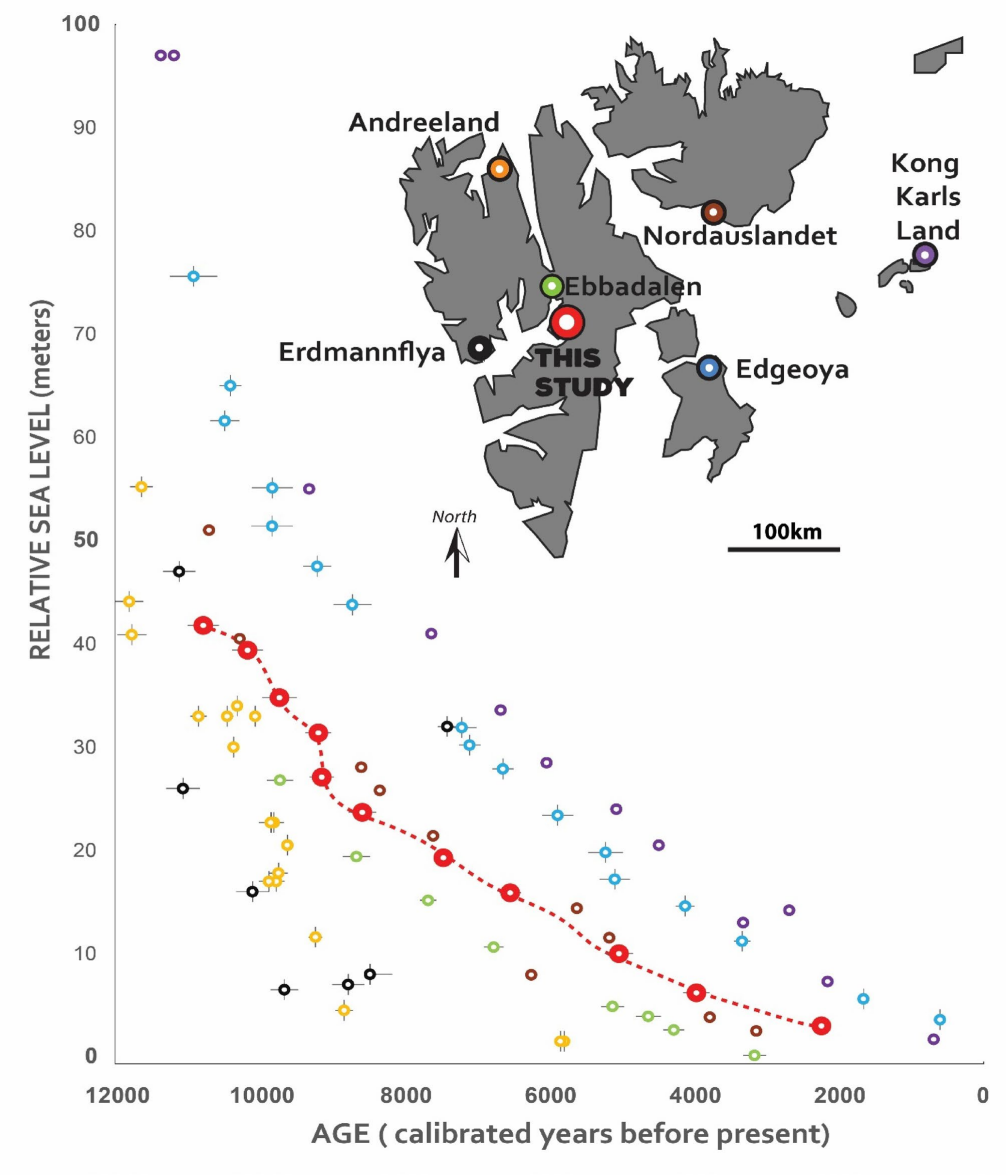
Holocene relative sea-level curves from the Svalbard Archipelago (for location see color coding in inset map). Sites include Andreeland^8^, Erdmannflya^56^, Billefjorden^18^; Edgoya^32^, Nordauslandet^27^, and Kong Karls Land^25^. Elevations of all sites were adjusted to the respective local datum using the height difference between mean tide level and the elevation of the modern berm as given in the original publications. The figure was created in Adobe Illustrator 2025 https://www.adobe.com).

Formation of the Bjonasletta beach-ridges correspond to a period of relative sea-level fall from 40 to 2 m asl (Fig. 3). This period is characterized by a period of near-linear fall by 10 m between 10.8 and 9.2 ka BP (resulting rate of fall is 6.3 mm yr^−1^), followed by a drop of 4 m in 100 years (rate 40 mm yr^−1^), associated with the last stage of rapid glacial unloading of the Svalbard-Barents Sea Ice Sheet^36^. Since then, there were 7000 years of slowly decelerating sea-level fall at an average rate of 3.4 mm yr^−1^. A similar trend was observed in nearby Sassendalen, which experienced a sea-level fall between 8 and 4 ka BP at a rate of 4.5 mm yr^[−157–58^. The apparent period of rapid fall after 9.2 ka BP may reflect geochronological uncertainties arising from the partial reworking of dated material. However, we regard this possibility as unlikely, as our ages are in chronological order and all samples selected for dating were well-preserved. Moreover, the artificial exclusion of any one age-control point from our sea-level curve still yields a period of overall rapid fall between 9.2 and 8.2 ka with a subsequent deceleration in long-term sea-level fall afterwards time. Thus, we infer that this period of rapid sea-level fall, followed by more slowly falling sea-level, is real and likely the result of accelerated glacio-isostatic adjustment. This is corroborated by recent studies from glaciated parts of the Arctic and Antarctic that show that rapid uplift (1.2–2.7 m in 100 years) driven by crustal unloading through glacier decay is possible^59,60^ and may have left traces in coastal architecture, such as non-erosive scarps in beach-ridge plains, characterized by the amalgamation of ridges^55^. Periods of accelerated land uplift due to glacio-isostatic rebound are also reported from the proximity to our study area: for example, in Grønfjorden, 70 km west of Bjonasletta, Farnsworth et al.^34^ suggested land uplift rates of up to 3 m in 100 years between 12.8 and 12.2 ka. Likewise, at Brøggerhalvøya, located 120 km northwest of Bjonasletta, Forman et al.^10^ reported rapid glacio-isostatic emergence around 9 ka BP with rates exceeding 2–3 m per century.

The pattern and rate of post-glacial emergence on Svalbard indicate that central Spitsbergen (this study^18^), northeastern Spitsbergen^61^; Nordaustlandet^27^, and eastern islands in the Barents Sea^25,32^, sustained the greatest glacier loads during the late Weichselian glaciation. During the same time, northwestern and southwestern Spitsbergen were peripheral to glacial loading and deglaciated earlier^30^. Consequently, all sea-level data from sites in eastern Svalbard plot above our new data from Bjonasletta. This pattern supports a comparatively thick Late Weichselian ice load over east Spitsbergen and the north Barents Sea area, as previously proposed by Elverhoi et al.^62^ and Lambeck^26^.

### Evolution of beach-ridge plain: insights into coastal processes

**Fig. 4:**
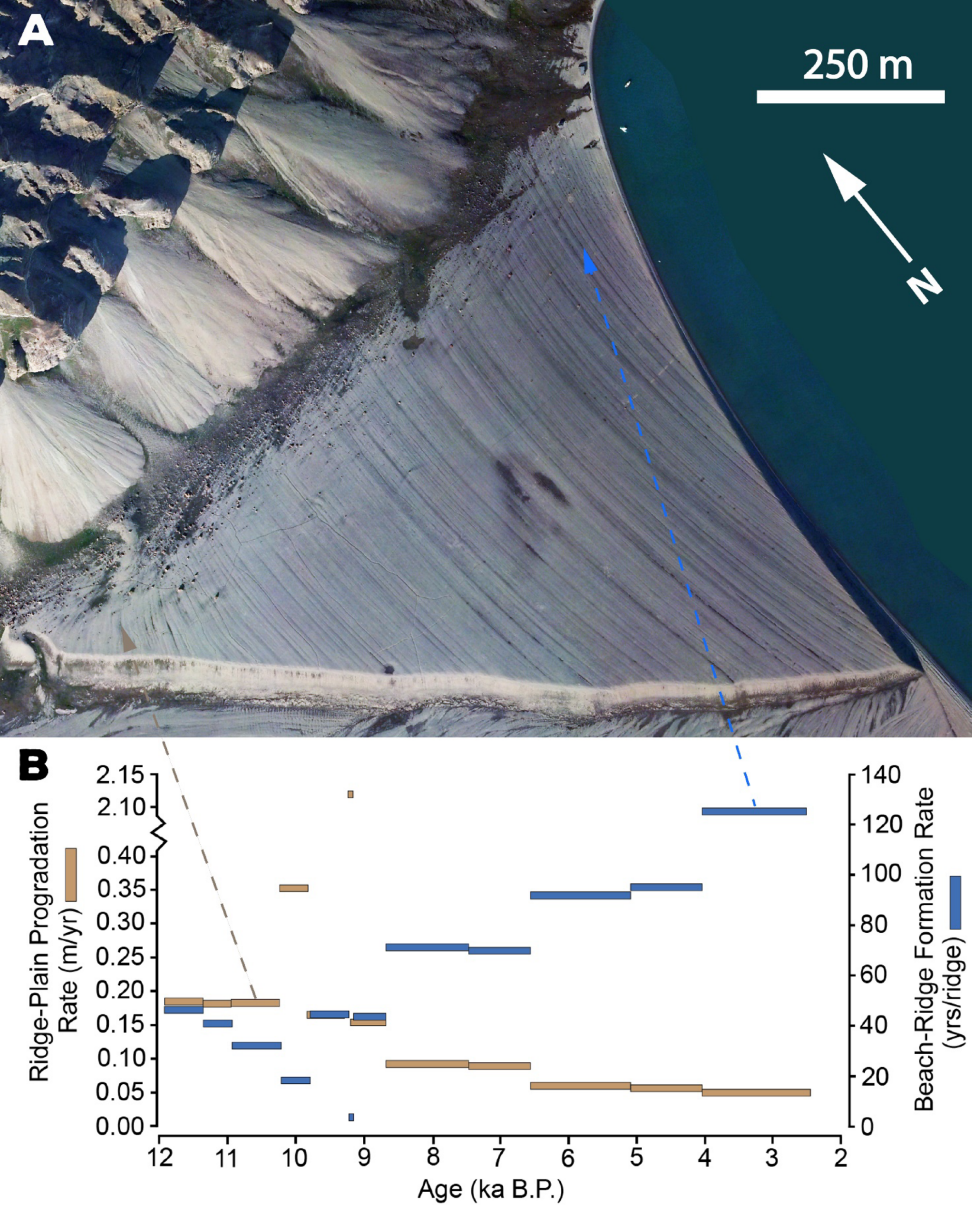
(**A**) Beach-ridges of the upper Bjonasletta beach-ridge plain. (**B**) Progradation rate and beach-ridge formation rate. The aerial photograph in the background is used with courtesy of the Norwegian Polar Institute. The figure was created in Adobe Illustrator 2025 https://www.adobe.com).

We calculated time-varying progradation rates of the at Bjonasletta beach-ridge plain (Fig. 3) to establish the first-ever reconstruction of Holocene coastal progradation in Svalbard. The analysis reveals a generally decreasing rate of progradation through time, from ~ 20 cm yr^−1^ ~10 ka to ~ 5 cm yr^−1^ by ~ 2.5 ka, which is strongly correlated with the rate of sea-level fall (linear regression r^2^ = 0.99, *p* < 0.0001; with removal of outlier high rate r^2^ = 0.71; *p* < 0.01); that is, faster sea-level fall leads to faster progradation in this normal-regressive system. Extrapolation of millennial progradation rates to the earliest (northwest ridges; elevation: ~45 m asl) and youngest (northeast ridges: elevation: 2.0 m asl) mapped ridges indicate that the beach-ridge plain of Bjonasletta prograded between 11.6 ka and 1.6 ka at an average rate of 10.9 m per 100 year (Fig. 3). These rates are far lower than those observed from lower latitude sandy beach-ridge systems, where average long-term progradation can vary by several orders of magnitude, and reach up to kilometers per century^63–68^. We also calculate rates of formation of individual ridges at Bjonasletta (Fig. 4). On average, one ridge formed every ~ 53 years over the course of the ~ 9500 years captured by our record. Ridge-formation rate generally decreased early in the record (reflected in Fig. 4 as fewer years per ridge) and then increased between 10 and 2 ka BP.

The rate of growth of beach-ridge plains, and the formation rate of individual ridges, are a function of sediment supply from alongshore and nearshore sediment sources, changes in available accommodation (sea-level trends, antecedent topography), and wave, wind, and storm climate^3,69–71^. At Bjonasletta, storm waves approaching from westerly directions are likely diffracted north around the tip of the beach-ridge plain, and therefore are not expected to act constructively in progradation as potential sediment sources are located at the northeastern termination of the beach plain, i.e. down-current of storm-induced longshore flows (rip currents). Waves of westerly storms might therefore be responsible for temporal sediment starvation and local erosion of the beach. As a result, storm waves are seen to be responsible for the genesis of the unconformities observed in the internal architecture of the beach sequence (Fig. 2) but are unlikely to foster construction of new beach-ridges.

In contrast, waves approaching the plain from easterly direction are highly likely to contribute to beach progradation by swash sedimentation. Synoptic meteorological data show that easterly to southerly winds are common in the study area, especially during the winter and spring seasons^39,40^. Katabatic winds coming from the glacier at the eastern end of Tempelfjorden represent another trigger of easterly waves, sustaining continuous swash sedimentation at the Bjonasletta beach, independent of geostrophic winds. These processes can aid in development of waves which allow for alongshore sediment transport (longshore drift) in the swash zone, the dominant longshore transport mode on gravel beaches^72^. This process potentially entrained sediment downdrift from the talus slopes of Templet and the large fan deltas northeast of the Bjonasletta beach plain, sustaining beach-plain progradation. Such patterns of alongshore sediment transport towards the Bjonasletta beach plain are likely at least within the modern system, as young spits at the fan delta points towards the Bjonasletta beach (Fig. 1B).

**Fig. 5:**
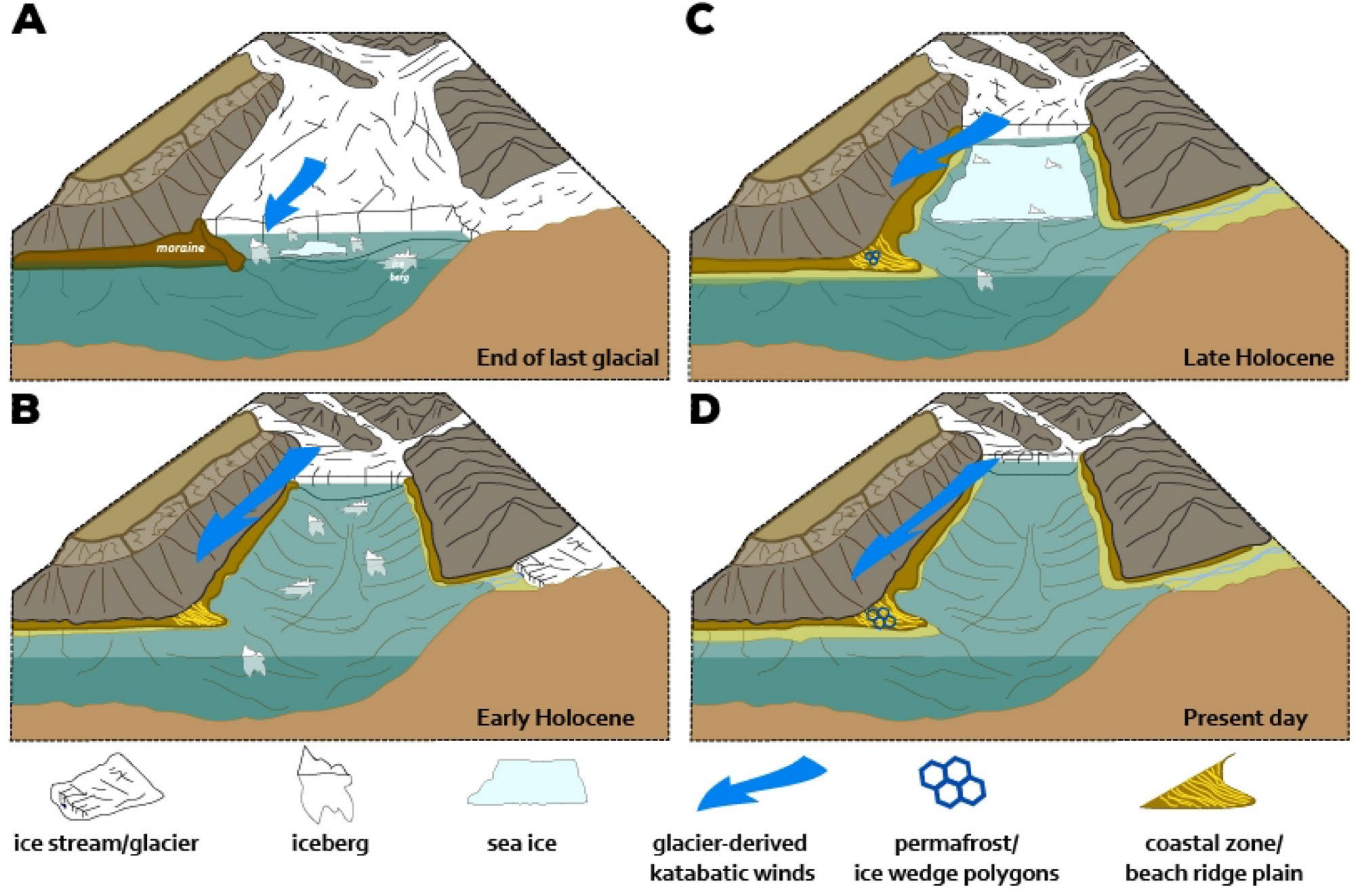
Stages of the evolution of the Bjonasletta coastal system, as controlled by the changes in glacial and sea-ice extent and sea-level changes. (**A**) Transformation of glacial landform (moraine) left along the fjord coast by retreating ice stream at the termination of the last glacial; (**B**) Early Holocene period of rapid sea-level fall, active slope processes in response to paraglacial transformation of fjord walls, long ice-free periods favoring wave activity and relatively long fetch between retreating front of marine-terminating glacier and evolving beach-ridge plain with wider and more morphologically diverse ridges; (**C**) Late Holocene, the advance of marine-terminating glacier front (shortening of wave fetch) together with slower relative sea-level fall and more stable sea-ice conditions limited wave action – formation of narrower beach-ridges, in the upper part of the plain permafrost conditions start to dissect beach surface; (**D**) Presently, due to glacio-isostatic uplift, the upper terrace is entirely cut off from marine processes, but beach formation is active along the lower (western) terrace. The figure was created in Adobe Illustrator 2025 https://www.adobe.com).

A primary factor influencing the height of wind waves is fetch^73^, i.e. the distance the wind interacts with the open-water surface. The maximal easterly fetch of the Bjonasletta beach corresponds to the ice-free length of Tempelfjorden, which hosts a marine terminating glacier, the Tunabreen, at its eastern end. Advance and retreat of this ice front can alter the fetch and thus the effective wave height at the Bjonasletta coastline during the open water season (Fig. 5). Available fetch for eastern Bjonasletta beach at present is around 13.5 km, and was within range of 1 km before ~ 11.2 ka ^38^, the time when beach-plain development likely started. A simple calculation of the effective wave height (see Methods and Materials) underlines the assumption that changes in fetch likely impacted sedimentation at the Bjonasletta beach during the Holocene (Fig. 5). A change in fetch from ca. 1 km (representing the onset of the Holocene, when the glacier front was close to the cape) to 13 km (representing current conditions, which are cooler than the Early to Mid-Holocene maximum retreat) would result in an approximate 300% increase in potential significant wave height at the Bjonasletta beach.

The shortening of the fetch by glacier advance is therefore expected to effectively reduce sediment fluxes and, by extension, the rate of beach plain progradation, on multi-decadal to centennial time scales (Fig. 4). Changes in fetch length may also impact the rate of ridge formation. For example, prevailing warmer conditions in the Early Holocene^74^ were likely conducive to the supply of sediments from nearby slopes and reworked glacial landforms to the prograding beach-ridge plain, thereby enabling the formation of ridges at a rate of approximately 45 years per ridge. The presence of extensive sea ice in the fjords and the regrowth of marine-terminating glaciers, which reduced fetch in the fjords and limited the transfer of wave energy onto the Bjonasletta beach, was enabled by climate cooling, particularly after 4 ka BP during the Neoglacial period. This resulted in a decrease in sediment mobilization at the shoreline (e.g. ^13,15,75^, which consequently led to a reduction in progradation, and elongation of the time required to build a given ridge to approximately 100 years (Fig. 4).

In summary, we conclude that the eastward growth of the Bjonasletta beach plain was governed by waves approaching from easterly directions. These waves not only triggered swash sedimentation at the beach but also sustained sediment supply by wave-induced longshore currents. The proposed combination of changes in glacial extent, sea-ice conditions, and sea-level, as captured in the Bjonasletta beach-ridge plain, are summarized in Fig. 5. Further investigations should focus on correlations between glacial advances and declines and the formation of new fjord shorelines to establish the conditions that facilitate wave development and coastal evolution^76–78^.

### Morphodynamics of the Bjonasletta beach-ridge plain: an archive of Holocene climate change?

Given its protected location (see Fig. 1), exposure only to easterly winds with temporally varying fetch, and its demonstrated construction through swash processes, the Bjonasletta beach-ridge plain holds potential to record changes in paleo-environmental conditions over the period of its formation. At the most fundamental, the rate of plain progradation is tightly linked to the rate of sea-level fall, which in Svalbard, in turn reflects the growth and decay of regional ice sheets and glaciers^36^. The spacing of individual ridges within beach-ridge plains such as Bjonasletta is commonly negatively correlated with the rate of progradation^79,80^, or the length of the embayment across which the ridge is building^68^. These trends reflect rapid abandonment of one ridge in favor of the next most seaward ridge, and the presumed equal distribution of a set sediment volume in a given period of time along the length of a plain to form a ridge, respectively. However, at Bjonasletta, ridge spacing ranges from ~ 2 to 12 m (avg: 6.7 m) — far tighter than in most beach-ridge plains globally (average 35–70 m^68^) — and shows no correlation with centennial-scale progradation rate (r^2^: 0.04), ridge-formation rate (r^2^: 0.04), or ridge length (r^2^: 0.05). That is, ridges that formed more quickly, or during a period of faster progradation, are spaced no more closely than those formed over longer periods of time. Likewise, the longest ridges are not significantly more closely spaced than the shortest ridges in the plain. Together, this indicates that ridge spacing is independent of sediment flux or plain progradation rate (or, by extension, the rate of sea-level fall). The amplitude of individual ridges at Bjonasletta (defined as the difference in height of a given ridge and its adjacent swale; see Methods and Materials) varied through time, from between < 1 cm to > 40 cm (avg: ~18 cm). This metric likewise shows no correlation with progradation rate (r^2^ < 0.01), ridge-formation rate (r^2^ < 0.01), or ridge length (r^2^: 0.05).

**Fig. 6:**
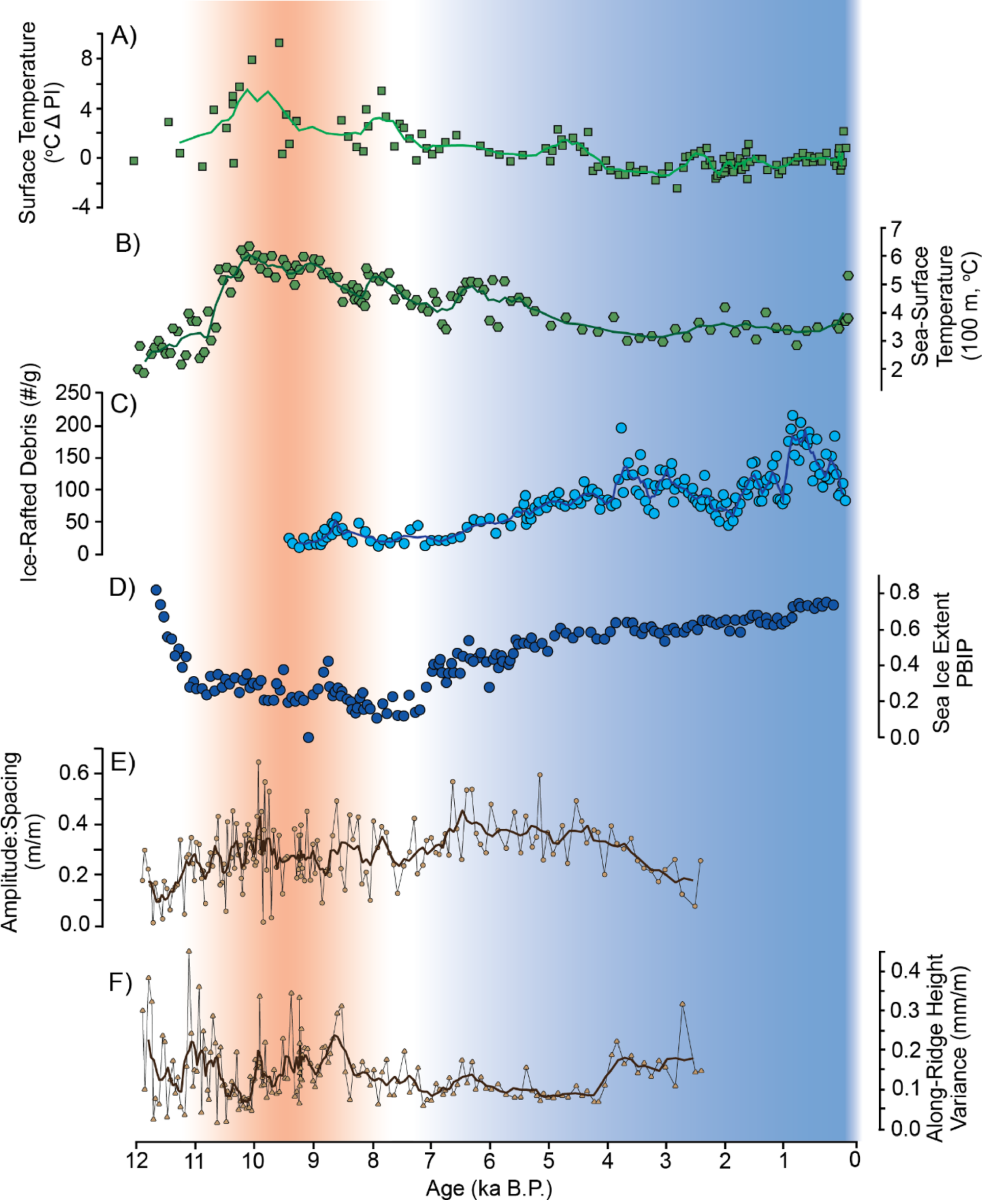
Record of regional climate signals within the Bjonasletta beach-ridge plain. Records include (**A**) UK_37_-derived western Spitsbergen land^48,52^ and (**B**) Fram Strait sea sub-surface temperatures^82^, (**C**) ice-rafted debris (IRD) and PBIP_25_-derived sea-ice extent^82,83^. Results of morphometric analyses reveal temporal trends in the amplitude: spacing relationship (**E**) and height variance along individual ridges (**F**) within the Bjonasletta beach-ridge plain. The figure was created in Adobe Illustrator 2025 https://www.adobe.com*).*

Independent from the factors controlling beach-ridge plain growth, changes in the ridge amplitude-to-spacing ratio largely mirror those observed in independent climate proxy records (Fig. 6). Specifically, amplitude: spacing is generally higher and more variable during relatively warm (Early Holocene) periods and lower, with muted variability, during cooler (Neoglacial) times (Fig. 6E). Ridge rugosity (internal variance in height of individual ridges) shows similar trends, peaking during the Early Holocene climate optimum between ~ 10–9 ka BP, but largely muted during cooler periods in the Middle to Late Holocene (Fig. 6F). We attribute these observed trends to temporal variability in wave energy associated with changing fetch in the Tempelfjorden basin to the east of the Bjonasletta ridge plain, stemming from changing glacier extent and sea-ice coverage. Specifically, stronger wave conditions are associated with higher and more complex runup patterns along gravel beaches^81^, a condition favoring higher, more widely spaced, and more topographically complex beach-ridges.

Together these insights allow the drawing of direct links between regional climate and sea-level changes and attendant patterns in progradation and beach-ridge morphology at Bjonasletta. In the Early Holocene (11.7–8.2 ka BP), a warmer climate favored ice-sheet melt, reduced sea-ice conditions^52,82,83^ and — possibly — stronger katabatic winds^84–88^ (Fig. 6A–D). Progradation of the Bjonasletta beach-ridge plain progressed at a linear rate during this period (Fig. 4B), reflecting gradual isostatic uplift as glaciers retreated.

Glacier retreat, combined with reduced sea ice and stronger winds, resulting in higher wave energy within Tempelfjorden and the development of ridges at Bjonasletta that were higher, more rugose, and more widely spaced (Fig. 6E, F). By ca. 9.2 ka, sea-level fall slowed and ridge progradation abruptly decelerated from ~ 17.7 to 6.6 m per 100 years (Figs. 3 and 4). This coincides with an episode of regional climate cooling linked to a North Atlantic freshwater event^52,89^ (Figs. 6A, B), followed by glacier re-advance in Tempelfjorden^38^, as independently captured in ice-rafted debris (IRD) and sea-ice proxies (Fig. 6C, D). Continued neoglacial cooling (Figs. 6A, B), glacier advance (Fig. 6C), and expansion of sea ice (Fig. 6D) in the Late Holocene would have reduced the easterly fetch within Tempelfjorden and associated wave energy available for mobilizing sediment, as well as reduced the amount of frozen ground (permafrost) that can bind berm material and hinder swash sediment transport^90^. The result was slowed progradation and the formation of lower, narrower ridges of more consistent alongshore height. Assuming a near-constant rate of isostatic adjustment (leading to the observed near-constant relative sea-level fall), the beach plain also became steeper during these times (Fig. 3). This reconstruction assumes that the ice and permafrost conditions that prevailed along the fjord coasts in the Late Holocene, along with the protected nature of the eastern Bjonasletta shoreline, combined to minimize the impact of regionally stormier weather that coincided with the onset of cooling of the climate at the end of Mid-Holocene^91^.

In conclusion, we find that the Bjonasletta beach plain presents a record of local sea-ice extent and wave climate as filtered through resulting beach-ridge morphology. Specifically, the low ridge-and-swale morphology of the beach plain reflects sub-centennial-scale variability in mean wave run-up height, whereas the variability of easterly fetch on time scales of millennia, paired with varying sea-ice extent, controlled the rate of beach progradation and the steepness of the beach plain. As such, Bjonasletta presents a novel example of the utility of coarse-grained, high-latitude beach-ridge systems as archives of regional climate change (specifically temperature and sea ice) and of attendant coastal-system responses.

## Conclusions

Arctic beach-ridges have long been valued as records of Holocene sea-level change. Here, we demonstrate that, under the right conditions, the sensitivity of these landforms to local wave energy allow them to also archive changes in regional climate. At the sheltered Bjonasletta beach-ridge plain, the availability of sediment from local slopes and enduring fair-weather conditions enabled the construction of the beach-ridges. This allowed the system to preserve an almost complete record of Holocene sea-level change and, independently, coastal evolution in response to changing wave conditions associated with temperature-driven changes in sea-ice and glacier extent, which in turn altered katabatic wind strength and the orientation and size of the fjord. As the glaciers advanced and the fjord shortened during the colder Neoglacial phase of the Holocene—a period characterised by stable ice conditions—the beaches that formed over the last 5000 years became lower and narrower. This contrasts with the wide and more morphologically complex ridge surfaces that formed during the warmer first half of the Holocene. We argue that beach progradation in Bjonasletta and other Arctic sites is linked with the duration of annual nearshore sea-ice coverage, which limits wave action at the beach.

In comparison with previous studies that have used the elevation of preserved beach-ridges solely to reconstruct relative sea-level changes, here we present a novel and nuanced approach to extracting signals of both paleoenvironmental change and attendant coastal response. Additionally, our sea-level curve serves to confirm the previously established Svalbard-Barents Sea Ice Sheet center; and a high-precision geochronology has enabled us to confirm the exceptionally rapid drop in sea-level at the dawn of the Holocene.

The core components of our approach—combining drone imagery/photogrammetry, ground-penetrating radar (GPR), and morphometric analysis with paleoclimate/sea-level data—can be successfully reproduced in other coastal environments, particularly those in the inner-fjord locations where beach-ridges are well-preserved. While our methodology is transferable, the specific results e.g., the shape of the relative sea-level curve or the rate of beach-ridge evolution) will necessarily be site-specific due to local controlling factors such as glacio-isostatic adjustment (GIA) history, sediment supply and storm frequency and intensity.

Further quantitative treatment of the amplitude, spacing, and height variability of wave-built gravel beach-ridges would additionally open new avenues for the use of these globally abundant formations as archives of paleoenvironmental change. Finally, we stress that although numerous studies of polar fjords have indicated the potential importance of katabatic winds in fjord ventilation, glacier retreat rates or sea ice distribution, their role in beach formation in cold climates has not yet been fully elucidated.

## Methods and materials

### Postglacial relative sea-level changes

**Fig. 7:**
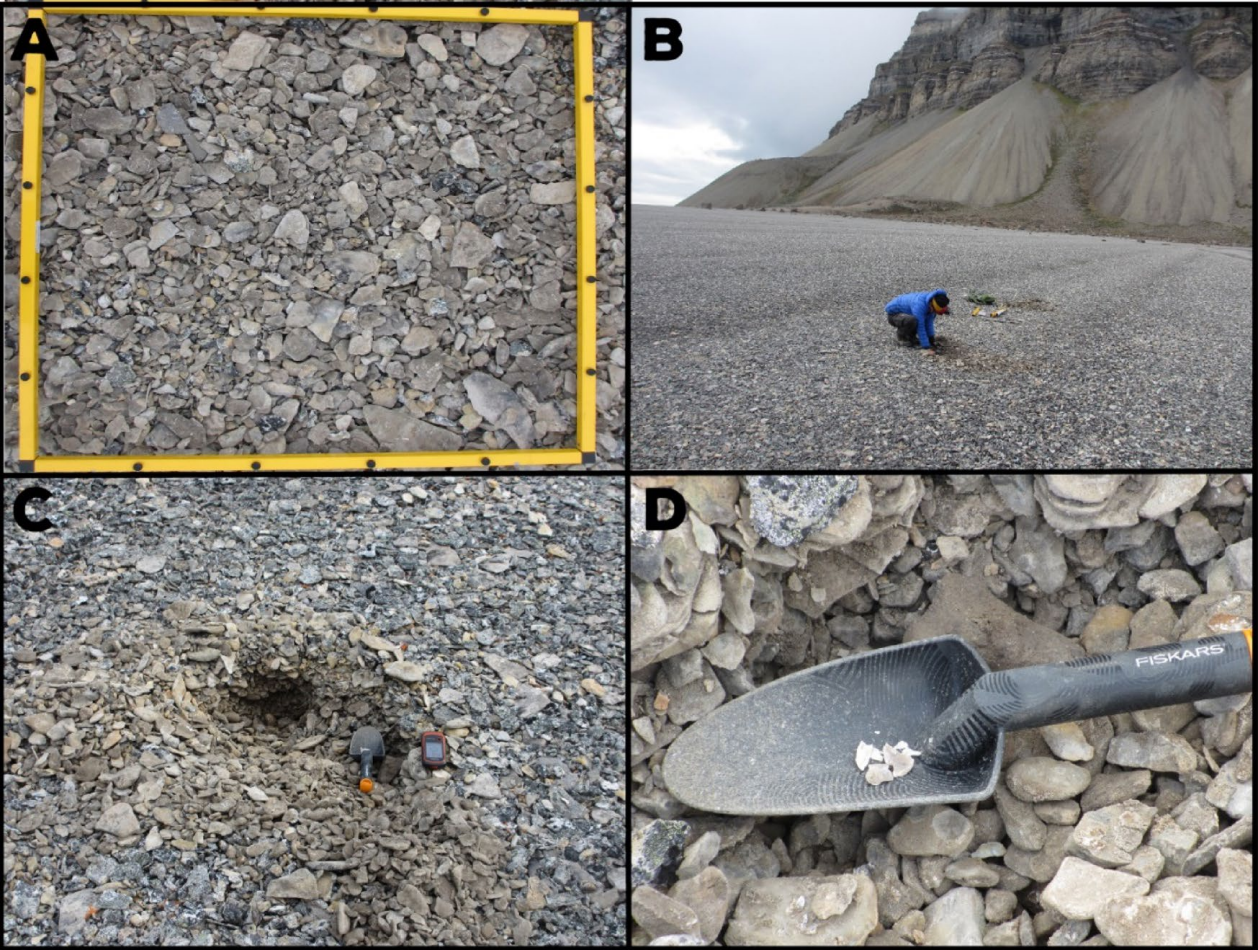
Mollusc shell sampling along Bjonsletta beach-ridge plain. (**A**) Surface of beach-ridge, yellow frame 0.5 × 0.5 m for scale (**B**) Careful digging through ridge crest using spatula and penknife, (**C**) most of the shells were found in the first 5 cm from the ridge crest, (**D**) full shell collected from site BJONA I1 – shell broke during collection, it was fully-preserved in the sediments. The figure was created in Adobe Illustrator 2025 https://www.adobe.com).

Following the approach of Long et al.^18^, we collected 11 samples for radiocarbon dating from near the crests of beach-ridges spanning the cross-shore width of the plain (Fig. 7). Of these, 10 were small, juvenile specimens of molluscs selected based on the degree of preservation (complete half shells with few signs of surface abrasion) to minimize the likelihood of reworking before deposition within the beach-ridges. Samples from higher ridges (Bjona_L1-F1) were dominated by *Astarte spp* arctic-boreal and shallow water species that occur in the Arctic and adjacent North Atlantic waters^92^. Within the crests of lower beach-ridges (Bjona E1-C1) we found *Mytilus spp.* shells, a common indicator of milder conditions in the Arctic associated with Atlantic Water influx^93^, that likely became locally extinct on Svalbard around 3.7 ky BP^94^. One sample (Bjona A1) was of well-preserved driftwood debris, fully embedded in beach gravel, and likely deposited in the upper intertidal zone as wrack. All specimens were removed from shallow (< 20 cm deep) pits dug into the surface of ridges and cleaned of inorganic or non-carbonate material in the lab.

Accelerator mass spectrometer (AMS) radiocarbon dating was conducted by Poznań Radiocarbon Lab (Table 1) following pretreatment (vacuum oxidation to CO_2_ for wood debris; hydrolysis with dilute HCl for shells). Resulting^14^C ages were calibrated using CALIB 8.1.0^95^ with either IntCal20 (wood debris)^96^ or Marine20 (*A. borealis* shells)^97^ calibration curves. A marine reservoir correction of -61 ± 37 yrs. was applied to all marine samples, following Pieńkowski et al.^98^. All dates presented in the text are calibrated, 2-sigma (2-σ) years before present (BP).

**Table 1:** Raw and calibrated radiocarbon dates for the Bjonsletta beach-ridge plain, Svalbard.

Sample ID	Lab accession #	Latitude	Longitude	Elevation (m asl)	Dated material	Reported age (^14^C yrs BP)	Cal. 2-σ age (yrs BP)*
Bjona_A1	Poz74582	78.39543	16.83686	1.4 ± 0.5	Driftwood	2220 ± 35	**2228 ± 103**
Bjona_C1	Poz74583	78.39498	16.83343	4.7 ± 0.5	*Mytilus edulis*	4100 ± 35	**4042 ± 204**
Bjona_D1	Poz74584	78.39432	16.83126	8.5 ± 0.5	*Mytilus edulis*	4905 ± 35	**5085 ± 203**
Bjona_E1	Poz74790	78.39375	16.82815	14.1 ± 0.5	*Mytilus edulis*	6245 ± 35	**6550 ± 185**
Bjona_F1	Poz74585	78.39279	16.82626	17.6 ± 0.5	*Astarte borealis*	7090 ± 35	**7452 ± 150**
Bjona_G1	Poz74586	78.39252	16.82181	22.5 ± 0.5	*Astarte borealis*	8250 ± 40	**8655 ± 227**
Bjona_H1	Poz74587	78.39226	16.81865	25.4 ± 0.5	*Astarte borealis*	8650 ± 40	**9189 ± 193**
Bjona_I1	Poz74588	78.39182	16.81471	29.7 ± 0.5	*Astarte borealis*	8690 ± 40	**9239 ± 193**
Bjona_J1	Poz74589	78.39215	16.81063	33.1 ± 0.5	*Astarte borealis*	9170 ± 50	**9815 ± 259**
Bjona_K1	Poz74591	78.39237	16.80564	37.3 ± 0.5	*Astarte borealis*	9470 ± 50	**10,230 ± 228**
Bjona_L1	Poz74592	78.39233	16.80121	40.1 ± 0.5	*Astarte borealis*	9980 ± 50	**10,937 ± 231**

*All dates in text are reported as 2-sigma median calibrated ages before 1950 (in bold) with error derived from full range of possible calibrated ages and incorporating instrument error.

All radiocarbon ages were calibrated using CALIB 8.1.0^95^. The single terrestrial sample (woody debris) was calibrated with the Intcal20 calibration curve^96^. Marine samples (all molluscs) were calibrated using Marine20 from Pieńkowski et al. (2022) corrected to a ΔR of Western Svalbard marine molluscs of − 61 ± 37 ^14^C yrs. All are reported as median 2-sigma calibrated ages, and errors represent the full range of possible calibrated ages, and incorporate instrument error. Sample elevations are calculated from RTK-GPS geoid height using local geoid-undulation of 32.5841 m and corrected using UAV-based digital terrain model.

Significance value bold.

### Ground-penetrating radar (GPR)

**Fig. 8:**
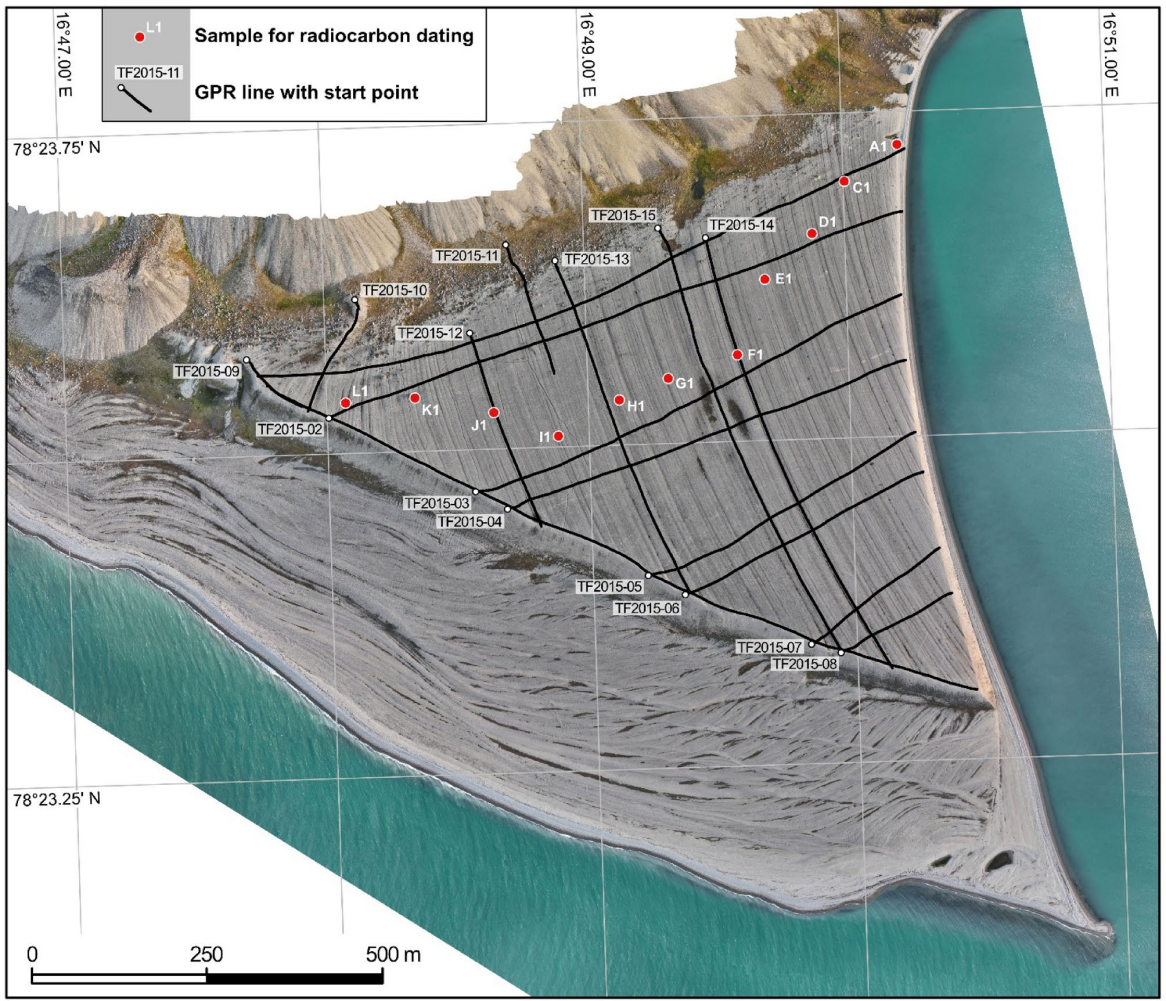
Ground-penetrating radar (GPR) data collection was done in discrete mode with a survey wheel as distance trigger. Trace increment was 0.05 m and manufacturer settings for pre-storage filtering were used. Along-profile topographic information was collected using a Leica GS09 GNSS receiver in real-time kinematic mode parallel to GPR measurements. The ortophotmap was constructed using aerial photographs delivered by Norwegian Polar Institute.

Information on beach-ridge geometry and sedimentary processes was obtained through stratigraphic analysis of GPR data collected using Geophysical Survey Systems Inc. (GSSI) SIR-3000 ground-penetrating radar with a 200 MHz antenna (see Fig. 8 for survey plan). Ground-penetrating radar is a non-invasive technique based on the transmission of high-frequency electromagnetic pulses. Signals are partly reflected at electromagnetic discontinuities in the subsurface and recorded as function of two-way travel time (TWT). The spatial resolution is controlled by the effective wavelength of the GPR signal and as such, GPR images an interference pattern controlled by the thickness of individual electromagnetically uniform sediment packages and the wavelength of the signal.

Data processing was done using the software package ReflexW^99^. Processing steps included static correction, dewow (removal of unwanted low-frequency component), frequency filtering, background removal, and gain correction. Topographic migration followed by correction for terrain morphology was applied to restore subsurface sediment geometries. The subsurface radar-wave velocity was estimated by fitting of diffraction hyperbolas. Resulting values range between 0.06 and 0.11 m ns^−1^, with higher velocities at greater depths; the median of 0.08 m ns^−1^ was used for time-depth conversion. Data interpretation follows the approach of radar-facies interpretation^100^ as applied by Lindhorst et al.^101^ and Lindhorst & Schutter^19^. The interpretation of unconformities is based on the tracing of reflection terminations and the identification of abrupt changes in the reflection pattern.

### Calculation of effective wave height

Our calculation of effective wave height aimed at a semi-quantitative estimation of the effect of changes in fetch length. Calculation is based on the equations provided by the Shore Protection Manual (Coastal Engineering Research Center 1984) and was done using the Java application provided by the US Geological Survey (https://csherwood-usgs.github.io/jsed/Fetch%20and%20Depth%20Limited%20Waves,%20USGS.html, accessed 2025-05-23). Input parameters comprise the wind speed 10 m above ground, the fetch length, and the water depth. We calculated the effective wave height for wind speeds of 25, 20, and 12 m s^− 1^ (corresponding to 10, 8, and 6 Beaufort). Maximum water depth in Tempelfjorden is about 30 m in the innermost Fjord, near the Tunabreen ice cliff, and about 100 m near the Bjonasletta Beach plain (nautical data from https://toposvalbard.npolar.no/, accessed 2025-05-23). For the calculation we therefore assumed an average water depth of 50 m. Calculated effective wave heights (rounded to 1 digit) for wind speeds of 25, 20, and 12 m s^− 1^ are (1) for 13.5 km fetch: 2.2, 1.7, 0.9 m; and, (2) for 1 km fetch: 0.6, 0.5, and 0.2 m.

### Unmanned aerial vehicle (UAV) and digital elevation model (DEM)

A DJI Mavic 3E drone equipped with RTK module and RTK base station was used to map the upper Bjonasletta beach-ridge plain. The survey was performed on 10 September 2023. The weather conditions were favourable for flight and mapping, with partly overcast sky, almost no wind and temperature around 0–2 °C. The survey covered approximately 3 km^2^ with 775 images taken from 100 m altitude and an average ground sampling distance of 5.7 cm. The flight was planned with 80% frontal and 70% side overlaps to achieve high spatial accuracy. The resulting orthophoto and Digital Elevation Model (DEM) have a horizontal accuracy of 0.056 m and 0.129 m vertical accuracy respectively. The DEM and ortophotomap was constructed in WebODM, API v. 1.5.3, Engine v. 3.5.6 hosted online by the University of Bergen. The ODM quality report is presented Supplementary Material 2.

DEM derived from the UAV data (initially referenced to ellipsoidal height) was corrected using a local geoid-undulation value of 32.5841 m. This correction was subsequently validated against a stable geodetic control point, NP141 (provided by the Norwegian Polar Institute), which has a known elevation of 13.076 m a.s.l. The resulting DEM elevation at the control point was confirmed to fit the known height within the reported vertical accuracy limits of the DEM.

### Beach-ridge morphometric analyses

#### Development of a topographic position index

A Topographic Position Index (TPI) was created from the raw DEM derived from the UAV mapping. The TPI measures the slope positions and landforms of a landscape by comparing the elevation of a central point (the processing cell) to the average elevation of its surrounding area, allowing for the identification of subtle topographic features such as ridges, valleys, and depressions, which are otherwise difficult to discern from the raw DEM^102^. In Bjonasletta, ridges are no taller than 0.5 m in height relative to their adjacent swales, making the TPI an ideal method to highlight those differences.

The values of TPIs are dependent on the neighborhood of cells used as inputs: the average elevation and distribution of the cells in a neighborhood serve as the values of comparison to the central processing cell. Positive values indicate a pixel is above the average elevation of its neighborhood (i.e., ridges and boulders). As a result, high TPI values are aligned with the parallel pattern of ridges visible on aerial imagery, allowing for confident visual identification of beach-ridge lines. Because of the slope of the plain (younger ridges are ~ 50 m lower in elevation than the oldest ridges), the neighborhood had to be small enough to prevent the slope from overriding the fine elevation differences between the ridges and adjacent swales. We thus apply a neighborhood size of 100 cells in width and 80 cells in height (approximately 5.1 m x 4.1 m), centered on the processing cell.

### Calculation of ridge geometries

**Fig. 9:**
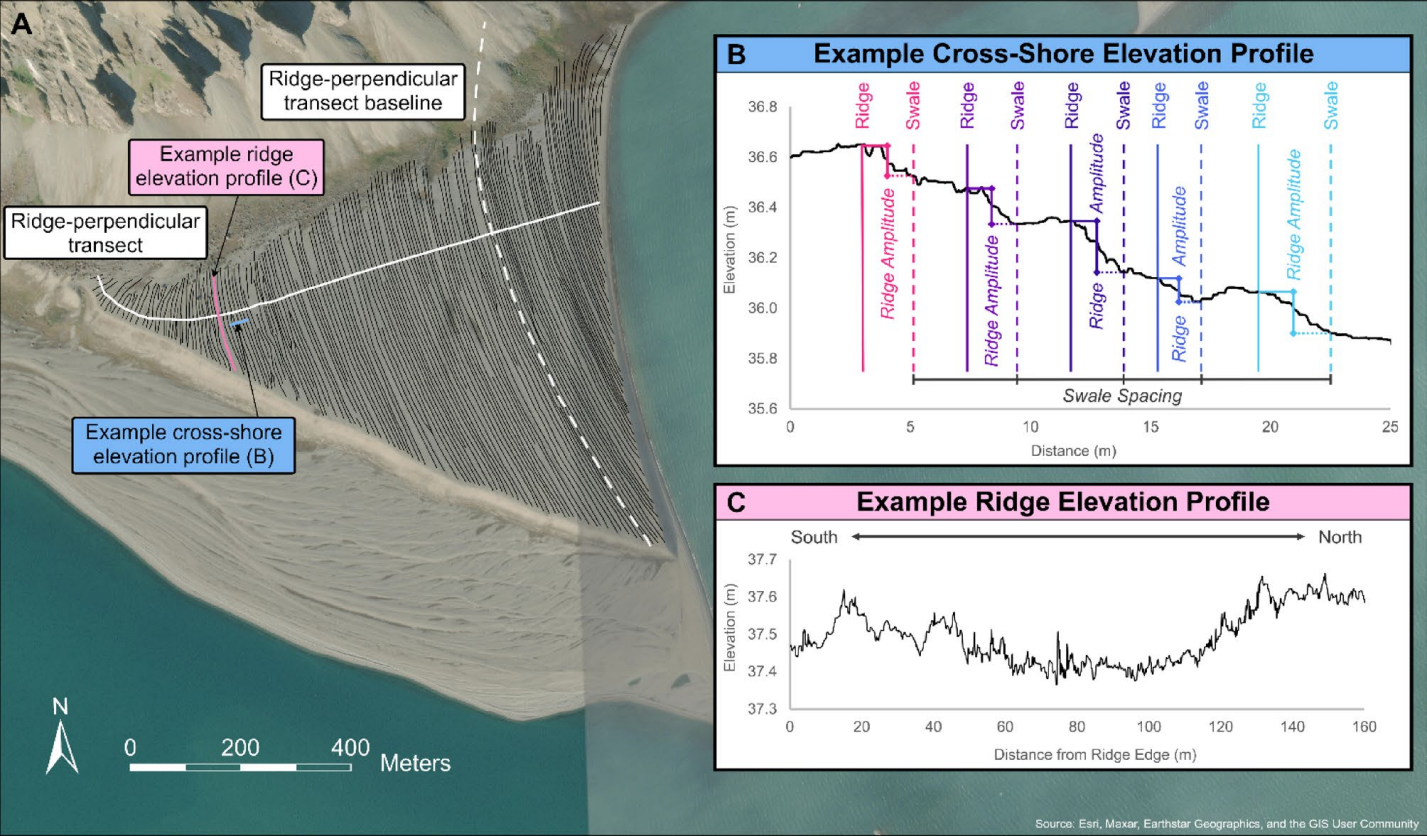
(**A**) Ridges identified on the Bjonasletta beach-ridge plain, showing one of the 41 ridge-perpendicular transects used to calculate ridge and swale spacing, and the baseline from which these transects were created; (**B**) Example cross-shore elevation profile of the Bjonasletta plain, identifying the approximate locations of five ridges and their paired seaward swales. Amplitude is calculated as the difference between the median elevation of a ridge and that of its paired swale, with spacing measured between adjacent swales; (**C**) Example along-shore elevation profile of a ridge, highlighting elevation variability across the Bjonasletta plain. The ortophotmap was constructed using aerial photographs delivered by Norwegian Polar Institute.

Elevation differences defining ridges are small enough to prevent automatic filtering of irregularities in the beach-ridge system, such as the presence of boulders and permafrost cracks. Therefore, ridges were manually identified as linear features characterized by high TPI values. This process resulted in a polyline layer consisting of 178 ridges across the plain (Fig. 9A). Each of these ridges was then assigned an age derived from spatial interpolation (and extrapolation, in the case of ridges which are older [younger] than our oldest [youngest] dated ridge) between dated ridges.

Ridges and swales were assigned a median elevation by sampling TPI values along the length of each ridge / swale at 0.2 m intervals. Ridge amplitudes were then calculated as the difference in those median values for each ridge paired with its associated swale, located to the immediate east (seaward direction) (Fig. 9B). In cases where a single swale did not adequately correspond to a ridge, a weighted average of two adjacent swales was employed. Ridge-perpendicular transects were then defined at 25 m spacing along a line drawn parallel to a central ridge and extending to the northern and southern boundaries of the plain. We calculated the spacing between adjacent ridges and swales along each of these 41 transects.

To calculate within-ridge height variance (Fig. 9C), we removed visible anomalies within the ridge system — boulders fallen from the adjacent cliff, kettles and depressions associated with melted ice, and edge erosion — to isolate potential patterns indicative of climate conditions affecting formation processes. We digitally segmented each ridge along the cross-shore transects used to calculate spacing, thereby greatly reducing variability in analyzed ridge length. We then detrended elevation values by calculating residuals from a linear model fit to each of the ridge segments. Segment variances were then calculated through these residuals, normalized by ridge length, and combined through a weighted sum to characterize overall ridge variability.

## Supplementary Information

Below is the link to the electronic supplementary material.

Supplementary Material 1

Supplementary Material 2

## Data Availability

We have uploaded DEM and ortophotomap to the Polish Polar Data Base repository, where the files can be accessed using the following https://polar.cenagis.edu.pl/dataset/digital-elevation-model-and-ortophotomap-bjonasletta-beach-ridge-system-tempelfjorden-svalbard
