# Supplementary material for: 10,000 years of centennially-resolved climate and sea-level change archived in Svalbard beach-ridge system
Source: Sci Rep. 2026 Jan 3;16:3627. doi: 10.1038/s41598-025-33652-w (PMC12847919; doi:10.1038/s41598-025-33652-w)
Supplement: Supplementary file 1 — Supplementary Material 1 [file 41598_2025_33652_MOESM1_ESM.pdf]

Supplementary Material 1

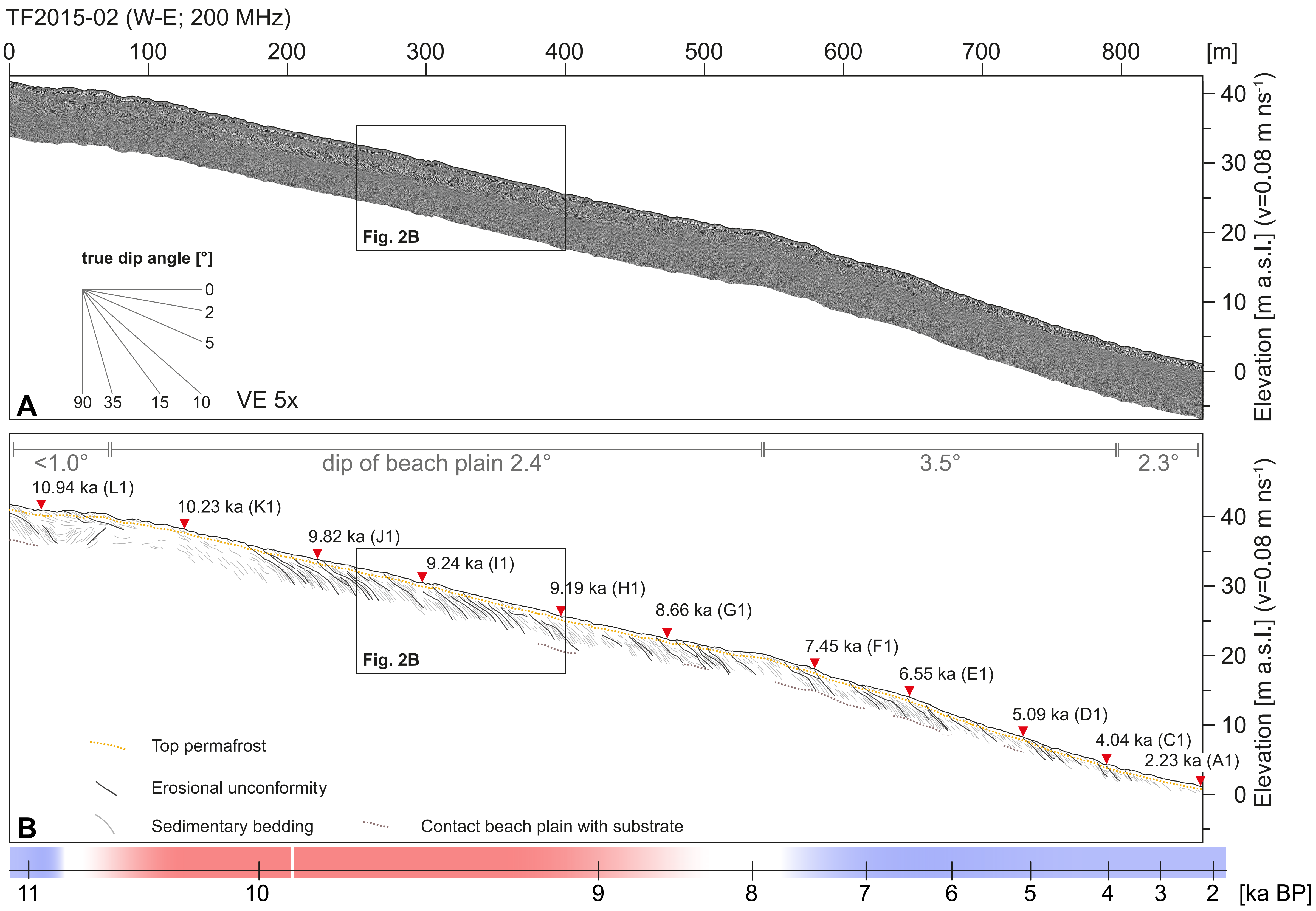

Ground-penetrating radar (GPR) line TF2015-02 (see Figs. 1, 8 for location).
A) GPR data obtained with a 200 MHz antenna (see Methods for details on data acquisition and processing). Data are shown with 5-time vertical exaggeration; **B)** Line-drawing and interpretation of A, Numbers A1 to L1 refer to calibrated radiocarbon ages (see Table 1 for data and Fig. 8 for sample positions) and were projected along the ridges onto the line. Yellow and blue bars indicate warm and cold climate episodes, respectively (compare Fig. 6)
