# Supplementary material for: 10,000 years of centennially-resolved climate and sea-level change archived in Svalbard beach-ridge system
Source: Sci Rep. 2026 Jan 3;16:3627. doi: 10.1038/s41598-025-33652-w (PMC12847919; doi:10.1038/s41598-025-33652-w)

Supplementary Material 2
ODM Quality Report

Processed with ODM version 3.2.0

Dataset Summary

Date	15/09/2023 at 16:21:38
Area Covered	3.040473 km²
Processing Time	4.0h:9.0m:23.0s
Capture Start	10/09/2023 at 06:52:07
Capture End	10/09/2023 at 07:42:27

Processing Summary

Reconstructed Images	758 over 775 shots (97.8%)
Reconstructed Points (Sparse)	914850 over 920333 points (99.4%)
Reconstructed Points (Dense)	34,557,012 points
Average Ground Sampling Distance (GSD)	5.7 cm
Detected Features	14,809 features
Reconstructed Features	8,809 features
Geographic Reference	GPS
GPS errors	0.07 meters

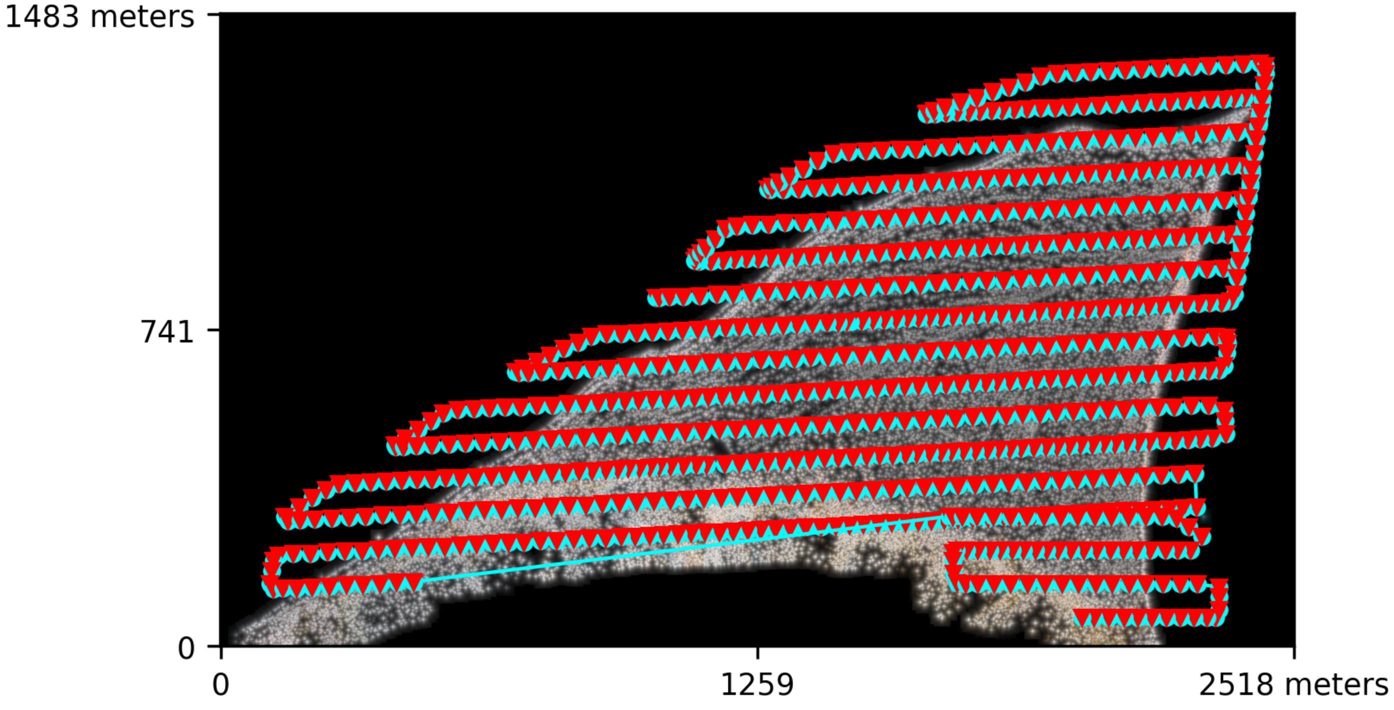

Previews

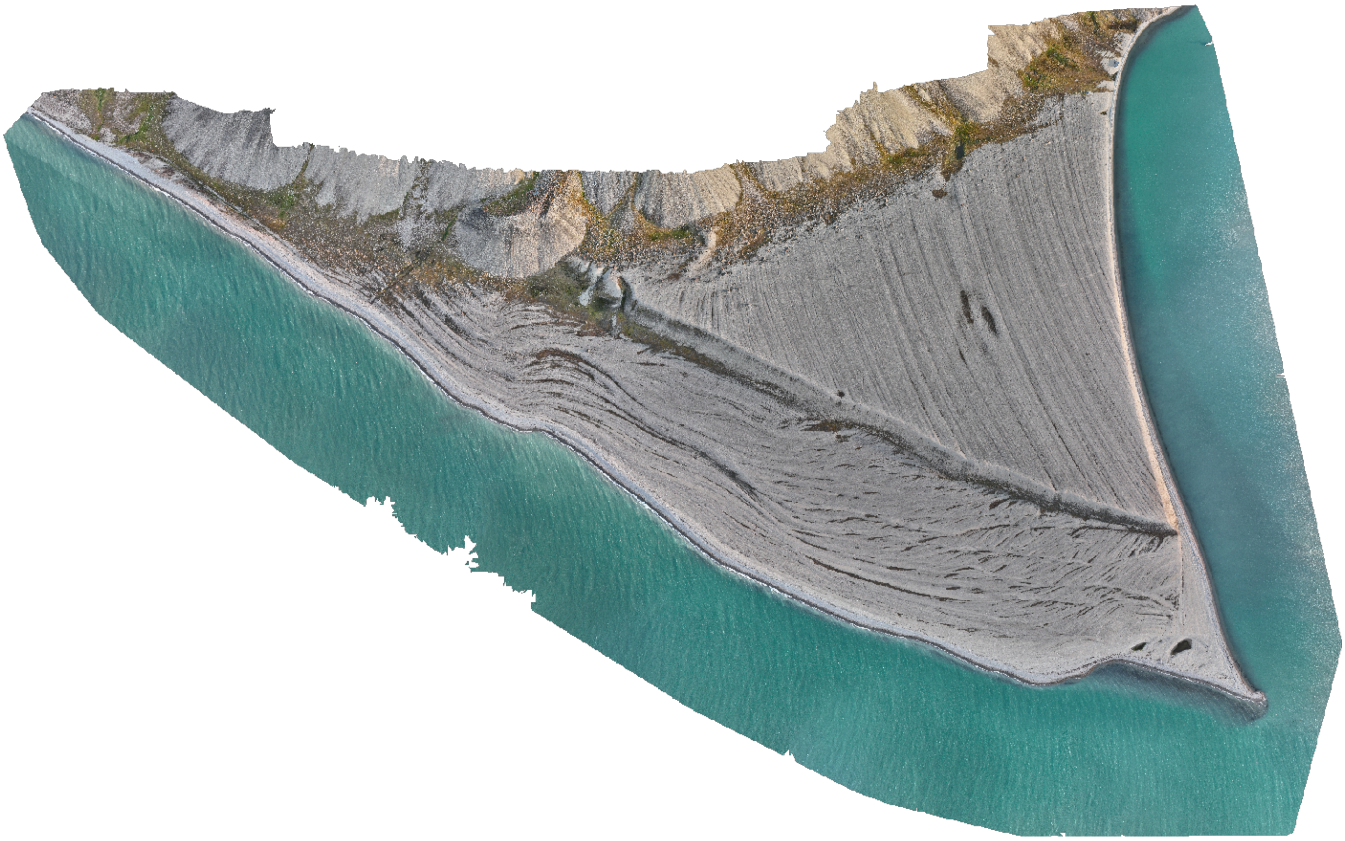

Orthophoto

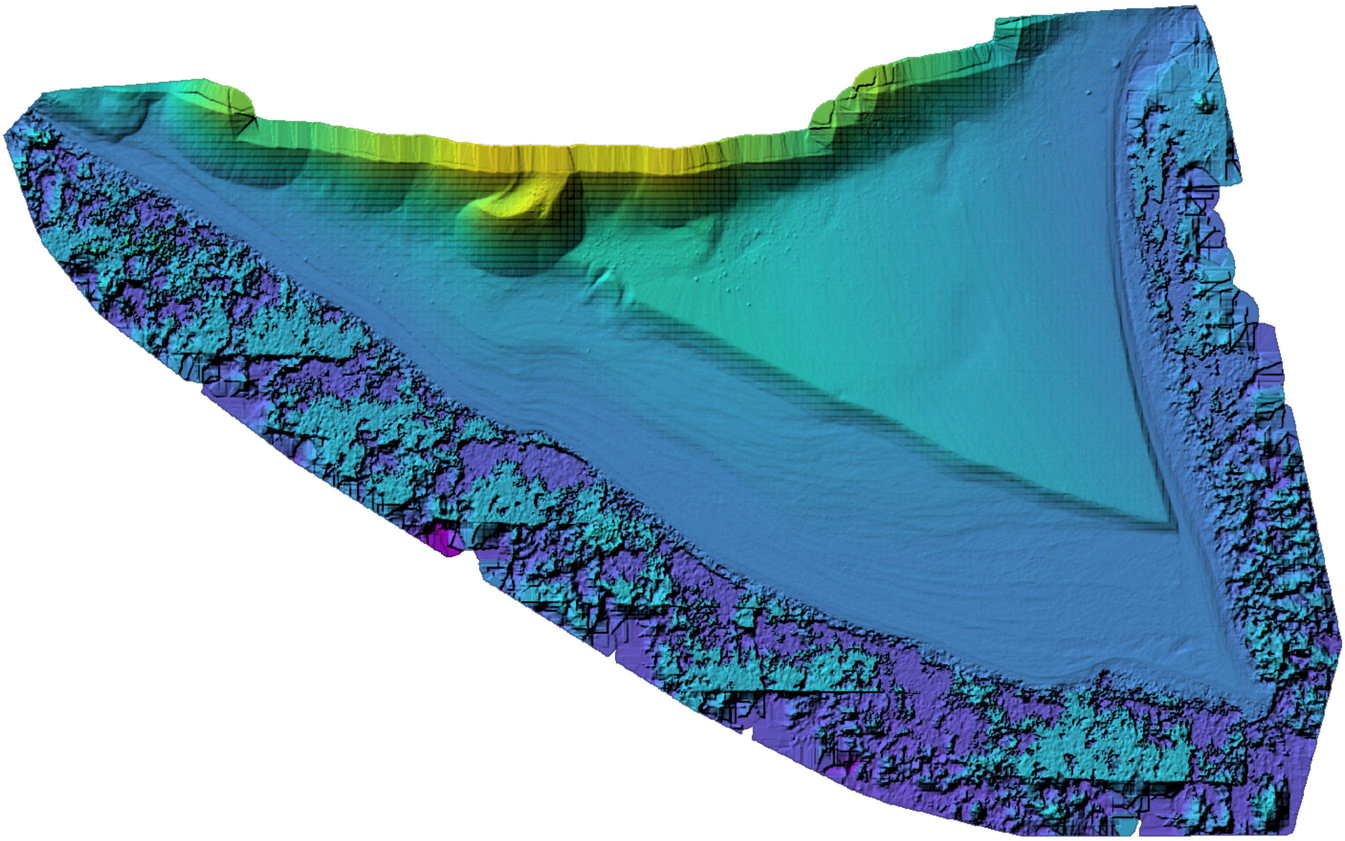

Digital Surface Model

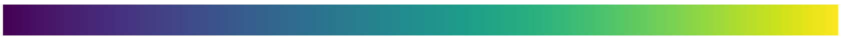

Survey Data

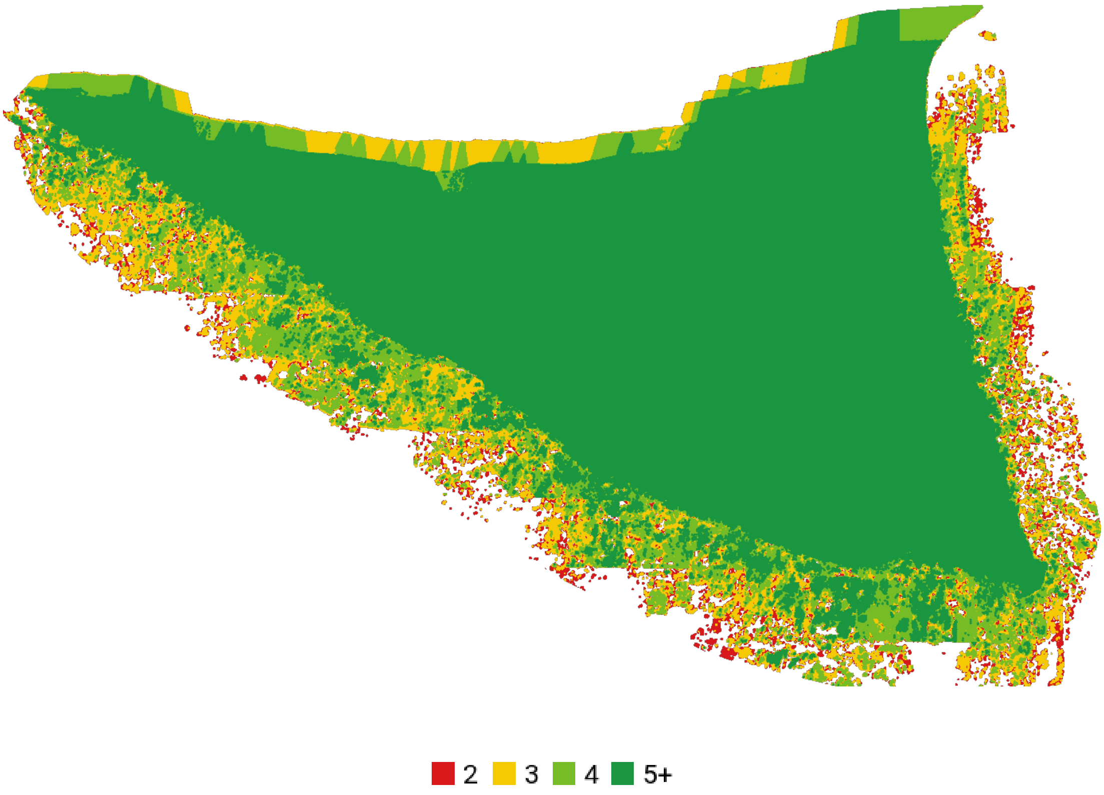

GPS/GCP/3D Errors Details

GPS	Mean	Standard Deviation	RMS Error
X Error (meters)	-0.000	0.024	0.024
Y Error (meters)	0.001	0.027	0.027
Z Error (meters)	-0.000	0.082	0.082
Total			0.067

3D	Mean	Standard Deviation	RMS Error
X Error (meters)	0.026	0.036	0.044
Y Error (meters)	0.032	0.040	0.052
Z Error (meters)	0.078	0.142	0.162
Total			0.094

	Absolute	Relative
Horizontal Accuracy CE90 (meters)	0.056	0.089
Vertical Accuracy LE90 (meters)	0.129	0.178

Features Details

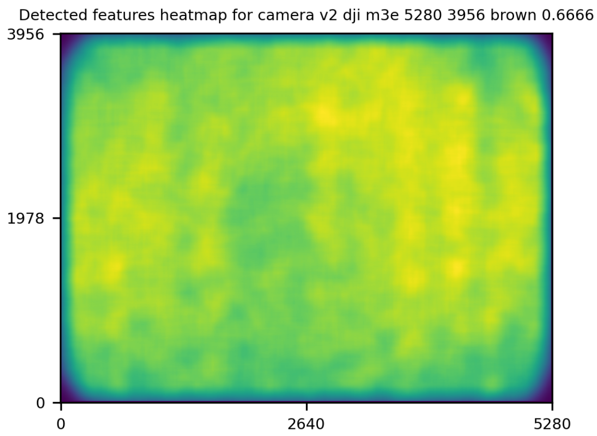

	Min.	Max.	Mean	Median
Detected	10011	15002	13604	14809
Reconstructed	47	12921	7803	8809

Reconstruction Details

Average Reprojection Error (normalized / pixels / angular)	0.10 / 0.58 / 0.00014
Average Track Length	6.47 images
Average Track Length (> 2)	8.49 images

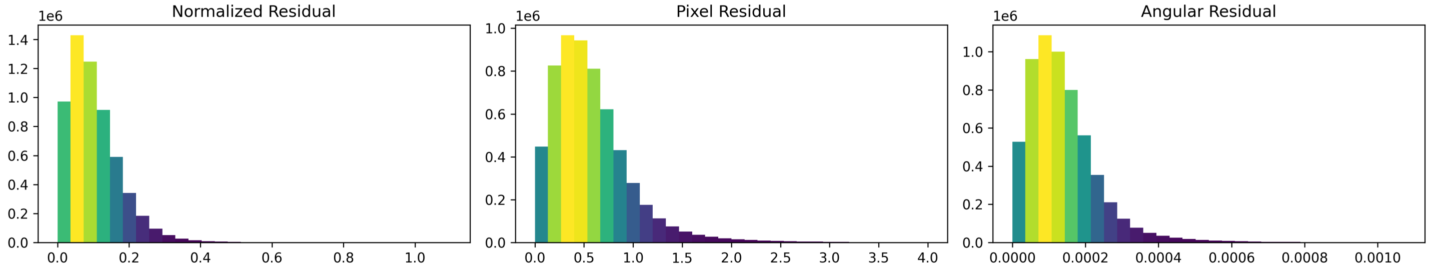

Tracks Details

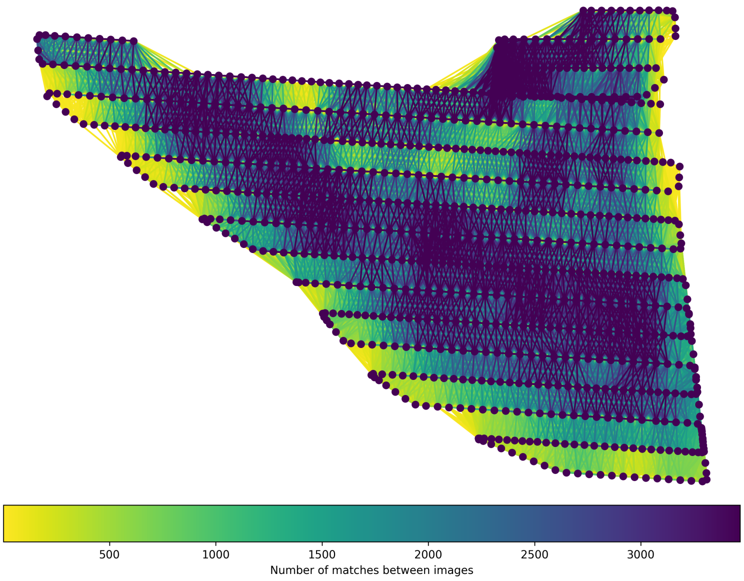

Length	2	3	4	5	6	7	8	9	10
Count	285670	140461	87585	61162	47132	37647	31358	26986	23229

Camera Models Details

v2 dji m3e 5280 3956 brown 0.6666

	k1	k2	k3	p1	p2	focal	aspect_ratio	cx	cy
Initial	0.0000	0.0000	0.0000	0.0000	0.0000	0.6667	1.0000	0.0000	0.0000
Optimized	-0.1076	0.0063	-0.0217	-0.0002	-0.0000	0.7010	1.0000	0.0008	-0.0004

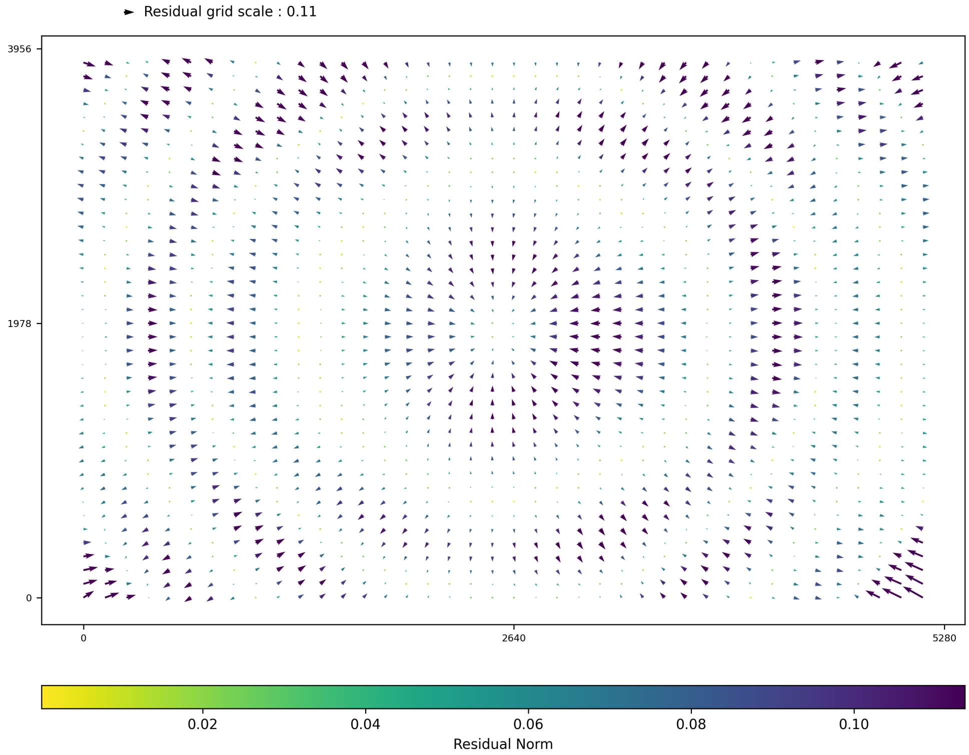

Supplement: Supplementary file 2 — Supplementary Material 2 [file 41598_2025_33652_MOESM2_ESM.pdf]
